# Neurobiological Models of Two-Choice Decision Making Can Be Reduced to a One-Dimensional Nonlinear Diffusion Equation

**DOI:** 10.1371/journal.pcbi.1000046

**Published:** 2008-03-28

**Authors:** Alex Roxin, Anders Ledberg

**Affiliations:** Computational Neuroscience, Department of Information and Communication Technologies, Universitat Pompeu Fabra, Barcelona, Spain; UFR Biomédicale de l'Université René Descartes, France

## Abstract

The response behaviors in many two-alternative choice tasks are well described by so-called sequential sampling models. In these models, the evidence for each one of the two alternatives accumulates over time until it reaches a threshold, at which point a response is made. At the neurophysiological level, single neuron data recorded while monkeys are engaged in two-alternative choice tasks are well described by winner-take-all network models in which the two choices are represented in the firing rates of separate populations of neurons. Here, we show that such nonlinear network models can generally be reduced to a one-dimensional nonlinear diffusion equation, which bears functional resemblance to standard sequential sampling models of behavior. This reduction gives the functional dependence of performance and reaction-times on external inputs in the original system, irrespective of the system details. What is more, the nonlinear diffusion equation can provide excellent fits to behavioral data from two-choice decision making tasks by varying these external inputs. This suggests that changes in behavior under various experimental conditions, e.g. changes in stimulus coherence or response deadline, are driven by internal modulation of afferent inputs to putative decision making circuits in the brain. For certain model systems one can analytically derive the nonlinear diffusion equation, thereby mapping the original system parameters onto the diffusion equation coefficients. Here, we illustrate this with three model systems including coupled rate equations and a network of spiking neurons.

## Author Summary

The brain holds a central position in scientific theories of rational behavior. For example, brain activity is thought to stand in a causal relation to the decision making behavior observed in two-choice perceptual discrimination tasks. Although a lot is known about both the brain activity and the response behavior during these tasks, the relationships between the two are not fully understood. In particular, how can one relate the high-dimensional dynamic activity of the brain to the low-dimensional descriptions of response behavior such as performance and reaction-times? Our approach to this question is to relate existing neurobiological models of brain activity to existing models of response behavior. In this paper we establish a formal link between standard, winner-take-all models of brain activity during two-choice tasks and a family of one-dimensional behavioral models known as diffusion models. Our analysis demonstrates a universal functional dependence between the external inputs to the neural populations in the neurobiological model on the one hand, and reaction times and performance in the one-dimensional model on the other. Importantly, we show that experimentally measured performance and reaction-times can be predicted through changes in these external inputs alone.

## Introduction

In perceptual two-choice decision making experiments one studies how sensory information influences response behavior. In each trial the experimental subject is presented with a stimulus and must use the information thus provided to choose one of two possible responses. The response behavior in these tasks, as defined by reaction times and performance, has been studied for over a hundred years [1]–[3] leading to a wealth of data and modeling results [4]. The reaction times are typically long compared to what would be expected based only on neuronal conduction times and vary considerably from trial to trial. Mean reaction times for error and correct trials are also, in general, found to be different. Moreover, subjects can be instructed to trade speed for accuracy. These facts are believed to reflect, at least in part, the decision making aspects of the tasks as opposed to sensory or motor aspects [1]–[5].

The aim of the work presented here is to account for the response behavior in two-choice decision making tasks in terms of the underlying neurobiology. In the remaining part of this section we will first describe one prominent family of behavioral models of response behavior, the sequential sampling models. Subsequently we will describe some neurophysiological findings, and models thereof, pertinent to our modeling framework.

### Behavioral Models of Two-Choice Decision Making

The response behavior in two-choice decision making tasks is well described by so-called sequential sampling models [6], of which Ratcliff's Diffusion model [7],[8] is a particularly successful variant. According to the Diffusion model there is a decision variable *X*, the evolution in time of which is given by the linear diffusion equation
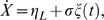
(1)where ξ is zero-mean Gaussian white noise with unit variance, σ is the noise strength and η*_L_* is a constant drift term. On each trial, *X* evolves from an initial condition until it reaches one of two fixed thresholds corresponding to the two decisions. The value of η*_L_* depends on the strength of the task-relevant information in the stimulus and is typically taken to be constant within a trial but allowed to vary between trials according to some distribution. The Diffusion model can account for many of the observed phenomena in reaction time tasks (e.g. [6],[9]). In particular, making the task easier corresponds to increasing the value of η*_L_* which leads to faster and more accurate decisions, e.g. [8]. The ability of a subject to trade speed for accuracy can be accounted for by changing the boundaries: by moving them closer to the starting points decisions become faster but less accurate [10]. Longer reaction times on error trials can be accounted for by introducing between-trial variability in the drift term, whereas shorter reaction times on error trials can be accounted for by considering a distribution of initial conditions [9].

The Diffusion model can be related to other behavioral models of decision making. It can be conceived of as a continuous-time version of random walk models (e.g., [11]). Usher and McClelland demonstrated how a two-dimensional connectionist model, with piecewise linear activation functions, can be reduced to a one dimensional diffusion equation [12]. The general form of the resulting diffusion equation differs from Equation 1 in that it includes a term linear in the decision variable and hence the resulting diffusion process is the so-called Ornstein-Uhlenbeck process. More recently Brown, Bogacz and co-workers have demonstrated that the linear diffusion equation can be derived from a range of different linear and piecewise linear connectionist and related models [13],[14].

### Neurophysiology of Two-Choice Decision Making and Models Thereof

Recently, neuroscientists have begun to investigate the single-cell neurophysiology of the decision making process in two-choice tasks (e.g., [15]–[19]). In many of the tasks used by neuroscientists the subject is presented a visual stimulus and the behavioral response is indicated by a rapid eye-movement (saccade) to one of two pre-specified targets. Decision making related neuronal activity in these tasks has been described in a number of brain areas that are known to be involved in the planning and control of eye-movements: lateral intraparietal area (LIP) [20],[21], dorsolateral prefrontal cortex [22], the frontal eye fields (FEF) [22], and the superior colliculi [23],[24]. There are several key features of the neuronal activity observed in these experiments that are important for our work: i) The average firing rate of cells in these areas is correlated to the response behavior of the animals. This indicates that the firing rate “represents […] the information on which the developing decision is made” [21]. Further support for this interpretation comes from a study showing a direct correspondence between the time-evolution of trial-averaged firing rates in the superior colliculus and the dynamics of the decision variable in the Diffusion model [25]. ii) The time-evolution of the trial-average firing rates is consistent with there being a competition between groups of cells associated with the two different decisions. The firing rate in the group associated with the correct decision shows, on average, an increase with time whereas the firing rate of the other group shows a decrease with time (e.g., [21]). Further evidence of a competition comes from microstimulation experiments [26]. iii) The neurons in many of the involved areas show evidence of nonlinear interactions. In particular, many cells continue to fire at an elevated rate after the stimulus indicating where to move the eyes to is removed [20]. This so-called persistent activity can be accounted for by models of recurrent networks of spiking neurons [27]. Indeed, in a series of papers, Wang and co-workers have shown that biophysically motivated cortical network models of a two-choice decision making task can qualitatively replicate some salient aspects of both behavioral and neurophysiological data [28]–[31]. Such models posit two populations of recurrently coupled excitatory neurons each of which receives input proportional to the relative evidence in favor of the choice which it encodes. The populations compete through interneuron-mediated inhibition leading to winner-take-all behavior. On each ‘trial’ the state of the system evolves until the activity of one of the two populations exceeds a fixed threshold indicating a decision for that choice. In this model, making the task easier corresponds to increasing the input to one of the populations relative to the other [28] whereas the speed-accuracy trade-off has been accounted for by adjusting the threshold [30].

### Relating Neurobiological Models to Behavioral Models

Assuming that the brain regions involved in the decision making process implement a winner-take-all strategy, as suggested by computational models, it remains unclear how this might lead to a response behavior best described by a one-dimensional diffusion processes. In other words, what is the relationship between the neuronal activity in putative decision making circuits and decision variables in behavioral models such as *X* in Equation 1 [32]? Recent theoretical work exploring the dynamics of winner-take-all models for decision-making has shown that several models can, in fact, be reduced to a one-dimensional diffusion process provided that the models themselves are linear. Usher and McClelland [12] studied a two variable connectionist model with inhibitory cross-coupling and linear-threshold activation functions. While the thresholding is nonlinear and leads to bistable behavior, the reduction to a one-dimensional diffusion process is possible only in the region where the argument of the linear-threshold function is the same for both variables and the dynamics, therefore, are linear. Brown et al. [13] study both linear and piecewise linear systems, while Bogacz et al. [14] study the relationships between a number of linear connectionist models and show under which conditions these models can be formally reduced to a one-dimensional linear diffusion equation. It remains however, unclear how the dynamics of such linear systems might be related to that of more biologically realistic neural models, which exhibit strong nonlinearities. A first step towards resolving this issue was taken by Wong and Wang [29] in which they derive a reduced system of coupled nonlinear equations from a full spiking network via a semi-analytical approach. They then show that the linearization of the reduced system in the unbiased case can be reduced to a one-dimensional diffusion equation at the point where the spontaneous state destabilizes ([29], Text S1). However, we may ask if the notion of a linear diffusion process is still valid once one takes into account nonlinear effects present in the system.

Here we show how one can go beyond linearizations to take into account nonlinear effects in neural winner-take-all models. In particular, we will show how models of neuronal dynamics in two-choice decision making tasks can be formally reduced to a one-dimensional nonlinear diffusion equation. Instead of focusing on a particular model system we consider a generic model of the neuronal dynamics with two key features: nonlinearity and competition. One obvious advantage of using such a general framework is that the extent of validity of the reduction is potentially very large. Moreover, most detailed models of the neuronal underpinnings of decision making do include nonlinearity and competition as important ingredients (e.g., [28],[33],[34]). Unlike in linear systems, a proper reduction of the dynamics to one dimension in nonlinear winner-take-all models leads to a nonlinear diffusion equation. The nonlinear diffusion equation takes the form of a stochastically driven normal form for an imperfect pitchfork bifurcation. The nonlinear diffusion equation not only provides the correct qualitative description of the dynamics in neural winner-take-all models in general, but can also be derived from model systems if they are analytically tractable. This allows one, in the context of two-choice decision making, to determine how the coefficients of this diffusion equation functionally depend on neurobiologically meaningful quantities.

## Results

Here we show how a winner-take-all model can be reduced to a nonlinear diffusion equation through general symmetry arguments alone. Despite the generality of the derivation, the resulting coefficients are directly related to biological meaningful parameters. We furthermore illustrate the correspondence of the nonlinear diffusion equation with neural winner-take-all models by deriving it directly from a system of coupled rate equations. Further examples, including a network of spiking neurons, are provided in the supporting material (Text S1). In using the nonlinear diffusion equation to calculate behaviorally meaningful quantities such as reaction-times and performance we must furthermore discuss the effect of initial conditions and the placement of thresholds. We will see that these are largely dictated by the type of dynamics seen in the actual winner-take-all models for which the nonlinear diffusion equation represents the correct asymptotic reduction. Finally, we show that the nonlinear diffusion equation can provide an excellent fit to behavioral data from the so-called random moving dot experiment.

### Derivation of the Nonlinear Diffusion Equation

We begin on a fairly technical note in order to provide some sense of the generality of the result. We will then make use of, for illustrative purposes, a simple model which nonetheless retains some biophysical plausibility . While the method we use may seem complicated and the algebra is, in general, involved, the idea behind the reduction is simple. We take advantage of the dramatic reduction in dimensionality which occurs spontaneously in dynamical systems near a point where the qualitative behavior of the system changes, i.e. stationary states appear, disappear or change in nature. Such transition points or bifurcations, are ubiquitous in physical and biological systems, e.g. see [35]–[37]. Here we make use of the fact that winner-take-all models for two-choice decision making, irrespective of their dimensionality or complexity, generically undergo such a bifurcation when two new stationary states appear, corresponding to the two potential ‘winner/loser’ pairs.

To emphasize the reduction in dimensionality we first consider a system of *n* nonlinear equations of the form
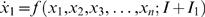
(2)

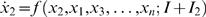
(3)

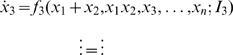
(4)

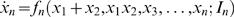
(5)where the *I*s are external inputs and *x_i_* represents the activity of the *i^th^* neuronal population, and 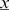 represents the time derivative of *x*. Here *x*_1_ and *x*_2_ are populations whose activity correlates with the two possible developing choices while the remaining populations are non-selective given the particular task. We note that for *I*_1_ = *I*_2_ the equations are invariant under the transformation (*x*_1_,*x*_2_)→(*x*_2_,*x*_1_), a property known as reflection symmetry. We see from this symmetry that the existence of the fixed point (*x*_1_,*x*_2_,…) = (*x_high_*,*x_low_*,…) implies the existence of the fixed point (*x*_1_, *x*_2_,…) = (*x_low_*, *x_high_*,…). That is, if there is state in which population 1 exhibits a high level of activity and population 2 a low level of activity, then we are assured the existence of the opposite state. If these are the only possible stable states at long times then Equations 2–5 constitute a so-called ‘winner-take-all’ system. Since we want the system to behave in a winner-take-all fashion only when provided with sufficient input, we furthermore assume the existence of a fixed point 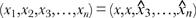, representing the spontaneous state. The derivation of the correct one-dimensional reduction of this system begins with the evaluation of the linear stability of this fixed point. We thus consider small perturbations of this state with the ansatz 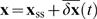 where **x**_SS_ are the steady state values and perturbations with growth rate λ have the form 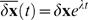. Plugging this ansatz into Equations 2–5 yields
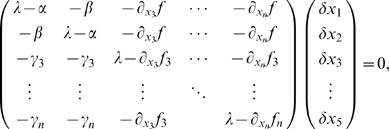
(6)where α is the derivative of *f* with respect to *x*_1_ in Equation 2 and *x*_2_ in Equation 3, β is the derivative of *f* with respect to *x*_2_ in Equation 2 and *x*_1_ in Equation 3, the γs are derivatives with respect to *x*_1_ and *x*_2_ and all derivatives are evaluated at the fixed point. It is clear that for α = β, which will occur only for a special parameter set, the first two rows cease to be linearly independent implying a zero eigenvalue with eigenvector **x**_cr_ = (1,−1,0,…,0) which corresponds to a mode for which either *x*_1_ or *x*_2_ increases while the other decreases, i.e. the winner-take-all dynamics we are interested in. If we wish our system to exhibit winner-take-all behavior then it must also be that the real part of the remaining *n*−1 eigenvalues is negative to avoid unwanted instabilities unrelated to the dynamics of interest and sufficiently distant from zero. This implies that perturbations along the eigenvector corresponding to the ‘winner-take-all’ instability neither decay nor grow linearly, while perturbations in any other direction quickly decay to zero. This is precisely the scenario in which a reduction to a one-dimensional dynamics is appropriate, as noted elsewhere, e.g [14],[29]. Specifically, the dynamics along *n*−1 of the *n* dimensions will rapidly converge from an initial state to a one-dimensional manifold along which the dynamics are slow. This separation of time-scales, where the time-scales are inversely related to the eigenvalues of the linearized system, is what gives us the reduction in dimensionality.

We now wish to derive an equation for the dynamics of the ‘winner-take-all’ instability. To do so we express the dynamical variables as **x** = **x**_SS_+**x**_cr_ *Y*(*T*)+---, where *Y* represents the slow dynamics along the critical eigenvector and *T* is a slow time scale. Note that the reflection symmetry of the system implies that the dynamics of *Y* should be invariant under the transformation *Y*→−*Y* since this switches the identity of *x*_1_ and *x*_2_. We assume that the increase in input, *I*, common to both *x*_1_ and *x*_2_ leads to the developing decision in the winner-take-all system and is thus the bifurcation parameter. This means that the linear growth rate of the spontaneous state must be proportional to the difference between the presynaptic input and the value of the input at the bifurcation although with an unknown prefactor, i.e. μ(*I*−*I*_cr_). The difference in inputs, *I*_1_−*I*_2_, breaks the reflection symmetry thereby introducing a constant term which, to first approximation, must be proportional to that difference although with an unknown prefactor, i.e. 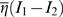. These two facts, coupled with the reflection symmetry, lead to the form of the equation describing the time evolution of *Y*

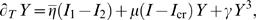
(7)where *I* = *I*_cr_ only when α = β identically, i.e. at point of instability, and ∂*_T_* is a time derivative with respect to the slow time *T*. Note that for *I*_1_−*I*_2_ the equation is invariant under *Y*→−*Y* as it should be (indeed, *Y* ^3^ is the lowest order nonlinearity which obeys reflection symmetry). The coefficients 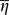, μ and γ can be calculated analytically from Equations 2–5. This line of argumentation can be made more exact mathematically, see Materials and Methods and the supporting material (Text S1) for examples from several systems, and is a standard technique in nonlinear dynamics known as multiple-scale analysis, see e.g., [38],[39]. For more complex systems which exhibit winner-take-all behavior, Equation 7 still captures the qualitative dynamics of the system near the bifurcation in general, although it may not be possible to calculate the coefficients. In addition, we are interested in the case of stochastically driven dynamics, which will lead, to leading order, to an additive noise term whose amplitude can also be calculated analytically for Equations 2–5, see Materials and Methods and supporting material (Text S1) for details. Finally, we arrive at the equation
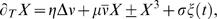
(8)where 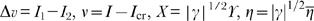 and the sign of γ determines the sign of the cubic term. We note here that although we have not derived Equation 8 from any particular system (and thus we do not know the functional dependence of η,μ, and σ on relevant physiological parameters), we nonetheless do know the leading order dependence on changes in external inputs to the two ‘competing’ populations. Thus the constant drift term is linear proportional to differences in these inputs while the linear term is proportional to the common input to both populations and is exactly equal to zero at the critical value. We also note that the evolution of *X* in Equation 8 can be thought of as the motion of an noise-driven, overdamped particle in a potential, or
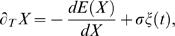
(9)

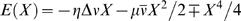
(10)The use of an analogy to an ‘energy landscape’ as an intuitive explanation for the dynamics in neural winner-take-all models is not new, e.g. [29]. However, here we have gone beyond analogy to show the actual form of the potential.

The framework we chose above for illustration, Equations 2–5, is a system of coupled nonlinear ordinary differential equations. However, the derivation of the nonlinear diffusion equation Equation 8 is not contingent on the original system having this particular form. For this reason we have chosen to illustrate the derivation of the nonlinear diffusion equation from three distinct model systems. Below we study a system of three coupled rate equations. In the supporting material (Text S1) we consider a system of three populations of integrate-and-fire neurons. Following the work of Brunel and Hakim [40] we recast the network as a system of coupled partial differential equations (Fokker-Planck equations) describing the evolution of the probability densities for the voltages. The nonlinear diffusion equation can then be derived from the system of partial differential equations. Finally, in the supporting material (Text S1) we also include a derivation from two coupled rate equations which Wong and Wang derived via semi-analytical arguments from a full spiking network [29].

### An Illustrative Example

We consider a simple model describing the activity of two excitatory populations of neurons which compete via a population of inhibitory interneurons. The equations are
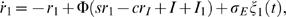
(11)

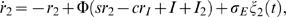
(12)

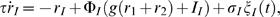
(13)where *r_I_*, *r*_1_ and *r*_2_ are the activity of the inhibitory and two excitatory populations respectively. The input to each population consists of a combination of recurrent and external inputs. For each excitatory population there is a recurrent excitatory coupling of strength *s*, an inhibition term with strength *c* and an input made up of a common and population specific parts, *I* and *I_i_* where *i* = 1,2. The inhibitory population receives excitatory drive from both populations with a strength *g* and input *I_I_* and we have neglected any self-inhibition term. The Φs are nonlinear transformations of the input. Fluctuations in the activity variables are expressed via unit variance Gaussian white noise terms ξ(t) with strength σ*_E_* and σ*_I_* for the excitatory and inhibitory populations respectively. Additionally, we have normalized time by the time constant of the excitatory populations and τ thus represents the ratio of the inhibitory to the excitatory time constant. For this system a ‘winner-take-all’ instability occurs for *s*Φ′(*I* = *I_cr_*) = 1. Following the general framework outlined above one carries out a multiple-scale analysis (see Materials and Methods) to arrive at Equation 8 with
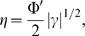
(14)

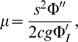
(15)

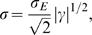
(16)

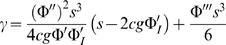
(17)where all derivatives of Φ are evaluated at *I* = *I_cr_*. Note that here the slow dynamics at the point of instability (bifurcation) are not dominated by the time constant τ (nor by 1) but rather by the near-zero eigenvalue of the critical mode. Thus extremely slow dynamics can thus be achieved in the vicinity of the bifurcation even in the absence of an intrinsic slow time constant such as that due to the activation of recurrent NMDA receptors, e.g. [28]. The presence of slow intrinsic time constants is, however, beneficial in eliminating oscillations and for obtaining realistic firing rates for working memory states, e.g. [28],[41].

Generically, two qualitatively different scenarios for winner-take-all dynamics can occur in nonlinear systems.

#### Supercritical

**Figure 1:**
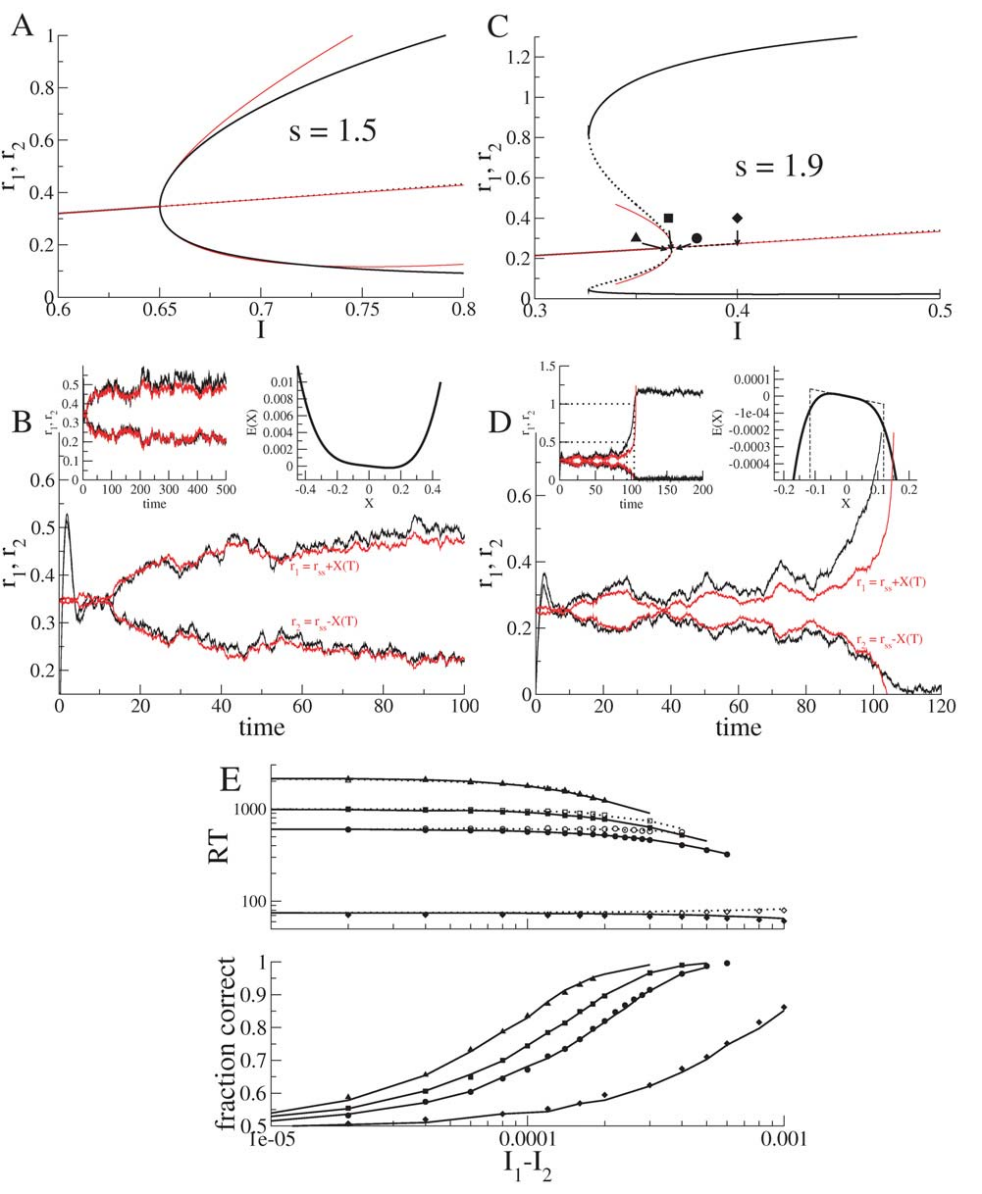
The bifurcation structure (A,C), typical dynamics (B,D) and behavioral measures (E) for the system of three coupled rate equations, Equations 11–13. For all panels shown, τ = *g* = *c* = 1 and *I_I_* = 0.2. The nonlinear transfer function is taken as 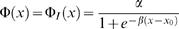 with α = 1.5, β = 2.5. (A) Below a critical value of the recurrent excitation the system exhibits a supercritical bifurcation. Shown are the fixed points of Equations 11–13 without noise using a Newton-Raphson solver, in black. Also shown are approximations of the fixed points given by Equations 8 with coefficients Equations 14–17, in red. Here *s* = 1.5 and *I*_1_ = *I*_2_ = 0. (B) Typical dynamics for a single trial given a supercritical bifurcation. Shown are the time-dependent variables *r*_1_ and *r*_2_ from integrating Equations 11–13 in black and the approximation obtained by integrating the nonlinear diffusion equation Equation 8 with coefficients Equations 14–17, in red. Left inset: the same trial shown for a longer time. Right inset: The energy function given the parameter values used for this trial. Here *s* = 1.5, *I* = *I_cr_* = 0.6502, *I*_1_ = 0.0025, *I*_2_ = −0.0025, σ*_E_* = σ*_I_* = 0.01. Initial conditions for rate equations, *r*_1_(0) = *r*_2_(0) = *r_I_*(0) = 0. Initial condition for nonlinear diffusion equation, *X*(0) = 0. (C) Above a critical value of recurrent excitation *s* the system exhibits a subcritical bifurcation. Lines are as in (A). Symbols show the value of the common inputs used in the four cases shown in (E). Here *s = *1.9 and *I*_1_ = *I*_2_ = 0. (D) Typical dynamics for a single trial given a subcritical bifurcation. Lines and insets are as in (C). Here *s* = 1.9, *I* = *I_cr_* = 0.3679, *I*_1_ = 0.001, *I*_2_ = −0.001. Initial conditions as for (B). (E) A comparison of the fraction of ‘correct decisions’ and mean reaction-times calculated by conducting simulations in the full system, Equations 11–13 (symbols), and with the nonlinear diffusion equation, Equation 8, with coefficients, Equations 14–17 (lines). The parameter values correspond to the bifurcation structure shown in (C). Different symbols indicate different values for the common input *I* and correspond to symbols in panel C. Specifically, *I*−*I_cr_* = −0.001 (triangles), 0 (squares), 0.0012 (circles), and 0.0321 (diamonds). Initial conditions for rate equations: *I* = 0.2 for *t* = −100 to *t* = 0 with appropriate steady state solutions. Initial condition for nonlinear diffusion equation *X*(0) = 0. Thresholds were 0.7 for the rate equation.

In the first scenario, the two ‘decision’ fixed points bifurcate continuously from the spontaneous state and therefore with small amplitude. Such a supercritical bifurcation can be seen in Figure 1A for Equations 11–13. The actual fixed points (black) as well as the prediction from Equation 8 with coefficients Equations 14–17 (red) are shown. Note that this scenario corresponds to a negative cubic coefficient in Equation 8. Systems of this type have been studied in the context of decision making, e.g. [42], by making linear and piecewise linear approximations. Figure 1B shows *r*_1_ and *r*_2_ (black) during a typical simulation in the supercritical case, as well as the rates predicted by Equation 8 (red), see the figure caption for parameter values. In comparing the original system and the nonlinear diffusion equation we must choose appropriate initial conditions. The derivation of the nonlinear diffusion equation itself assumes strongly attracting dynamics in all directions except along the slow manifold whose dynamics are described precisely by Equation 8. Assuming symmetric initial conditions in the original system (pre-stimulus), an infinitely fast approach to the slow manifold with stimulus onset would lead to *X*(0)∼0. The further from the bifurcation one is the worse this approximation will be. Nonetheless, assuming a fast transient after stimulus onset, the choice of *X*(0) = 0 is the weakest assumption possible. As an example of the insensitivity of the system response on initial conditions, see the fast oscillatory transient in Figure 1B (black) after stimulus onset (here *r*_1_ = *r*_2_ = *r*_I_ = 0) after which the dynamics converges rapidly to that predicted by the nonlinear diffusion equation (red). If we wish to treat the system as a model of decision making in the supercritical regime we must choose the appropriate placement for the thresholds. This has been studied elsewhere, e.g. [42]. We would only note here that in the regime where the system dynamics behave locally as a linear diffusion process, i.e. at the bifurcation point, the thresholds must be placed very near the spontaneous state. This is reflected in the U-shaped potential (Figure 1B, righthand inset) which strongly constrains the dynamics. For larger values of the common input the potential is a double-well and the dynamics near the spontaneous state are strongly repelling.

#### Subcritical

A positive cubic coefficient in Equation 8 indicates a subcritical bifurcation. In this case, the fixed points corresponding to a decision appear in a saddle-node bifurcation already below the critical input at the pitchfork bifurcation. These solution branches therefore already have finite amplitude at this point. Such a situation is shown in Figure 1C. In models representing the activity of neuronal populations a crucial variable in determining the criticality of the bifurcation is the recurrent excitation [27]. Indeed, this can be clearly seen in Equation 17, the sign of which clearly switches at a critical value of the recurrent excitatory coupling *s*. (For *s* near this critical value, the coefficient of the cubic term can be considered asymptotically small, justifying the addition of a quintic term, see Text S1 for details.) Strong recurrent excitation therefore naturally leads to a subcritical bifurcation and hence hysteresis. Network models describing the activity of neuronal populations in two-choice decision-making tasks have made use of this working-memory property which allows for a decision to be made and then ‘held in mind’, e.g. [28],[29]. This is additionally consistent with single-unit recordings of cells in area LIP of monkeys which exhibit delay activity [20],[21]. The subcritical case is therefore a more likely candidate for a neuronal instantiation of winner-take-all dynamics for this two-choice decision making task in LIP than the supercritical one. The dynamics in this case are qualitatively different from the supercritical case as can be seen in Figure 1D (initial conditions are as before). Here the firing rates (black) initially separate over a slow time scale. At some point this separation speeds up and the system quickly approaches a ‘decision’ fixed point. This acceleration of the separation is captured by a positive cubic coefficient in Equation 8, leading again to a rapid rise in the predicted rates (red). Unlike in the supercritical case, it is clear that reaction-times calculated by simulation of Equations 11–13 in the subcritical case will be relatively insensitive to the exact placement of a threshold as long as it is not too close to the spontaneous state. This is illustrated by the ‘thresholds’ shown by dotted lines in the left inset of Figure 1D. Here a twofold increase in the threshold leads to less than a 10% increase in the reaction-time. This difference clearly becomes more negligible the higher the thresholds are taken to be. This insensitivity to the threshold placement is captured in the nonlinear diffusion equation by the positive cubic coefficient which causes *X* to go to infinity in finite time, a consequence of an inverted-U potential, see right inset Figure 1D. It is interesting to note that the linear diffusion equation with fixed thresholds can also be expressed as the motion of an overdamped particle in a potential. In this case the potential also has an inverted-U shape although the sides of the potential have infinite slope as illustrated by the hypothetical potential shown by the dotted lines in the right inset of Figure 1D. Therefore the dynamics of nonlinear winner-take-all models near a subcritical bifurcation although qualitatively different than that of a linear diffusion process, nonetheless share some qualitative features. In particular, the same nonlinearities which lead to delay activity act to shape an effective inverted-U potential, thereby endowing the system with an intrinsic ‘soft’ threshold. This would eliminate the need for fine-tuning in the threshold-setting mechanism used by any downstream ‘readout’ neurons [30],[43]. We note, however, that one could choose to set thresholds near the spontaneous state, in which case the exact placement will strongly affect reaction-times and performance, as in for example [30]. Finally, Figure 1E shows a comparison of relevant behavioral measures calculated from simulations of Equations 11–13 and from the nonlinear diffusion equation Equation 8 with coefficients Equations 14–17. We will discuss the dependence of performance and reaction-times on the coefficients of the nonlinear diffusion equation in the next section.

### Performance and Reaction Time in the Nonlinear Diffusion Equation

**Figure 2:**
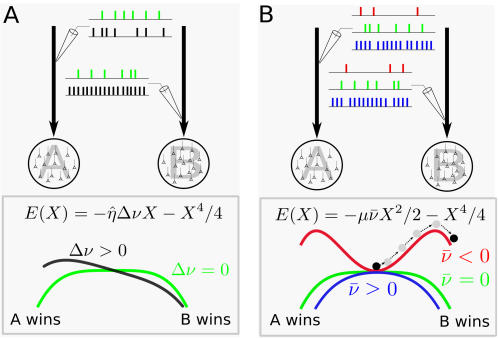
Diagram of the energy *E*(*X*) as a function of the difference in inputs Δν and the mean input 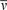 to two populations in a winner-take-all network. The populations are shown schematically as circles and the respective inputs as arrows. The relative level of input to the populations is represented by the spike trains “recorded” from the input arrows. (A) The energy *E*(*X*) as a function of the difference in inputs Δν shown for 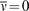. Two cases are shown. Case 1, in green: both populations receive the same average input. This results in a symmetric energy function. Case 2, in black: population B receives more input than population A. This tilts the energy function, biasing the probability of choosing population B over A. (B) The energy *E*(*X*) as a function of the mean input 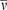 shown for Δν = 0 (both populations receive the same mean input). Three cases are shown. Case 1, in green: 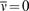. This results in a relatively flat energy function with zero curvature at zero. Case 2, in red: 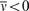. This results in a local minimum in the energy function. The system must escape over one of the two barriers for a decision to be made. Case 3, in blue: 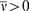. Here the inputs are large enough to transform the local minimum to a local maximum in the energy, making decisions faster and less accurate.

The explicit dependence of the coefficients in Equation 8 on the inputs to the two populations allows us to directly relate modulations in these inputs to changes in reaction-times and performance. In doing so we will make use of the formulation of a nonlinear diffusion equation as the motion of a particle in a potential, Equations 9–10, see Figure 2. An increase in the difference of the two external inputs tilts the potential in favor of the population with the greatest input, an effect also seen in linear, connectionist models, e.g. [12]. Modulations of the input common to both populations affect the curvature of the potential. For common inputs below the bifurcation, the potential exhibits a dimple, reflecting an attracting spontaneous state, while above the bifurcation the spontaneous state is repelling. This modulation of the potential via changes in the common input is a consequence of the nonlinearity of the system. In linear systems, changes in the mean input, given a fixed threshold, shift the position of the effective threshold for the decision making process, e.g.[14].

Below, we discuss these effects in greater detail, making use of exact expressions for reaction times *RT*(*X*_0_) and performance *P*(*X*_0_) as a function of the initial condition *X*_0_. See supporting material (Text S1) for the expressions.

### How Changes in the Difference in Inputs, Δν, Affect Reaction-Times and Performance

When both populations receive the same mean input, i.e. Δν = 0, the energy function, Equation 10, is symmetric, leading to an equal probability of escape through either boundary, i.e. performance *P*(0) = 0.5, see Figure 2A green. If one population receives more input than the other, i.e. its activity encodes the ‘correct’ choice, Δν≠0 and the probability of making the corresponding choice will be greater than chance, *P*(0)>0.5. This is reflected in the asymmetry of the energy function, which is now tilted towards the correct choice, see Figure 2A black. Reaction-times for the correct choice decrease monotonically with increasing Δν. Reaction-times are, in general, different for the error choice with respect to the correct choice for a fixed value of Δν and can exhibit non-monotonic dependence on Δν, see supporting material (Text S1). Mean reaction-times for error trials can, in fact, be slower or faster than those for correct trials depending on the value of the common input 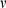. Performance increases with increasing Δν, owing to a more pronounced asymmetry in the energy. Indeed, it can be shown analytically that 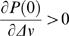, see supporting material (Text S1). An analogous effect is obtained in the linear diffusion equation, Equation 1, by changing the constant drift term.

### How Changes in the Input Common to Both Populations, 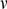, Affect Reaction-Times and Performance

Changes in the input common to both populations affect the quadratic term in *E*(*X*) through 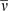. For 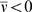 the spontaneous state is stable, reflected in the local minimum of the energy shown in Figure 2B red. As the common inputs are increased, 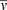 increases, destabilizing the spontaneous state and thus converting the local minimum to a local maximum for 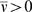, Figure 2B blue. For 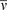 identically equal to zero, the spontaneous state is marginally stable, resulting in an energy function with zero curvature locally, Figure 2B green. Indeed, in this regime the dynamics in the vicinity of the spontaneous state behave similarly to those seen in the linear diffusion equation Equation 1, whose energy function is given by *E*(*X*) = −η*_L_X* with absorbing boundaries. Since reaction-times are given by the time it takes for the system to escape from the spontaneous state, it is clear that reaction-times decrease with increasing 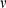 as the spontaneous state changes from attracting (local minimum) to repelling (local maximum). In fact, it can be shown analytically that 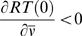 for Δν = 0, showing that reaction-times strictly decrease with increasing common input. Numerical investigation show that this holds also for Δν≠0 (not shown). Furthermore, it can be shown analytically that 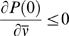 for any value of Δν, see supporting material (Text S1). The fact that both reaction-times and performance decrease monotonically with increasing common input suggests a novel mechanism to explain the physiological underpinnings of the speed-accuracy trade-off. That is, Equation 8 predicts that increases in common input to the two populations will lead to faster reactions and poorer performance while decreases in input will lead to slower reactions and better performance.

We note that increasing the noise amplitude σ in Equation 8 leads to decreasing performance. Increasing noise amplitude also tends to reduce reaction-times given initial conditions in the vicinity of the spontaneous state.

### Fits to Behavioral Data

To reiterate, Figure 2 shows how the energy landscape and hence the system dynamics in two-population winner-take-all networks is affected by changes in afferent inputs alone. This is important since the dependence on the input holds for all models irrespective of the details, while changes in the coefficients η, μ, and σ imply changes in single-cell and network properties specific to the model chosen.

We now show that such changes in input are sufficient to describe behavioral data in two-choice decision making tasks. Specifically, we consider data from two separate studies using the so-called random moving dots task, namely from Roitman and Shadlen [21] and Palmer et al. [10]. The coherence of the stimulus in these experiments is defined as the fraction of dots moving in one of two possible directions, the remaining dots moving randomly. The subject must indicate the direction of the coherent motion with a saccade. We choose this particular task as behavior and electrophysiologal activity have been well characterized [10],[20],[21], and biologically motivated two-population winner-take-all models have been evoked to describe the decision making process [28]–[30]. In particular, it has been shown that the stimulus coherence is encoded approximately linearly in the firing rates of direction-selective cells in area MT [44]. Evidence suggests that output from MT cells then drives neurons in area LIP whose trial-averaged activity is consistent both with the notion of a linear integrator (ramping activity with increasing slope for increasing coherence) and with that of competing populations of neurons (activity ramps up or down depending on whether the receptive field is in the preferred or anti-preferred direction of motion respectively) [20],[21].

**Figure 3:**
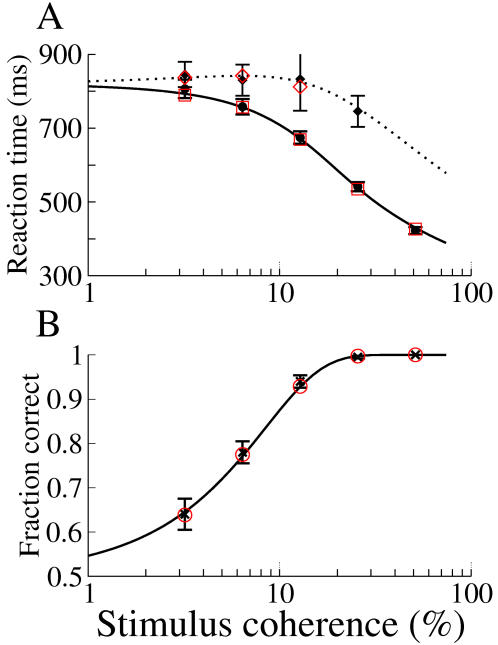
Fit to data from Roitman and Shadlen ([21], Table 2) with the nonlinear diffusion equation (Equation 8) and subsequent match with system of rate equations. Black symbols: experimental data. Error bars represent approximate 95% confidence intervals. Lines: solution of Equation 8. Red symbols: simulated data from a system of rate equations (Equations 11–13). (A) Reaction times on correct and error trials as a function of coherence. Circles (solid line) and diamonds (dotted line) are for correct and error trials respectively. (B) Fraction of correct responses as a function of coherence. Experimental data are shown as crosses. Parameter values for Equation 8: ηΔν = *coherence*×6.6667*e*−6, 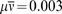, and σ = 0.00135. A fixed offset of 230 ms is added to the reaction times. We choose parameter values in the system of rate equations to yield precisely these parameters in Equation 8. See Materials and Methods for values.

**Figure 4:**
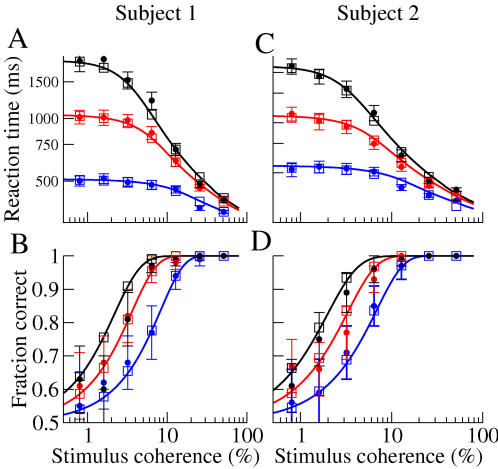
Fit to data from Figure 7 in Palmer, Huk, and Shadlen [10] with Equation 8 and subsequent match with system of rate equations. Filled circles: experimental data. Lines: solution of nonlinear diffusion equation, Equation 8. Open squares: system of rate equations. Error bars represents approximate 95% confidence intervals. Data are from three sets of experiments in which subjects are instructed to respond with 0.5 s (blue) 1 s (red) and 2 s (black). (A) Reaction times on correct trials as a function of coherence for subject 1. (B): Fraction of correct responses as a function of coherence for subject 1. Parameter values from Equation 8 for subject 1: ηΔν = *coherence*×1.25*e*−5, σ = 0.00135, 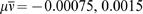, and 0.012 for 2 s, 1 s, and 0.5 s trials, respectively. (C) Reaction times on correct trials as a function of coherence for subject 2. (D) Fraction of correct responses as a function of coherence for subject 2. Parameter values from Equation 8 for subject 2: ηΔν = *coherence*×1.25*e*−5, σ = 0.00135, 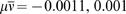, and 0.008 for 2 s, 1 s, and 0.5 s trials, respectively. A fixed offset of 260 ms and 220 ms was added to *RT* for subjects 1 and 2, respectively. We choose parameter values in the system of rate equations to yield precisely these parameters in Equation 8. See Materials and Methods for values.

In light of these experimental observations, it is reasonable to assume that the difference in inputs from MT cells to the putative neuronal populations in LIP which encode the two possible directions, increases linearly with increasing coherence. Therefore, we assume a linear dependence of Δ*v* on the stimulus coherence in Equation 8. Doing so provides an excellent fit to behavioral data, capturing performance as well as both correct and error reaction times, without having to vary any additional parameters, see Figures 3 and 4, solid line fit to symbols with error bars. Note that the difference in reaction-times for correct and error trials comes about due to the nonlinearity of the energy function which sits along the slow manifold (mean correct and error reaction times are identical given a linear energy function). This is in contrast to the mechanism evoked in [29] to explain longer error reaction times using a two-component system of rate equations. There they argued that asymmetries in the phase plane lead to trajectories for error trials which stayed closer to the stable manifold as they approached the unstable manifold. The trajectories therefore came closer to the saddle-point leading to long residence times before escaping. This mechanism relies on the full two-dimensionality of the system coming into play. It is likely that the mechanism we describe here is dominant near the bifurcation, while far from the bifurcation the full dimensionality of the system being studied must be taken into account in order to explain longer error reaction times.

We now show that the trade-off between speed and accuracy, commonly observed in reaction-time experiments [4], can be explained through changes in the common input to the two populations. The data shown in Figure 4 are from three sets of experiments in which human subjects are told to respond within 0.5, 1 and 2 seconds, and are shown in blue, red and black symbols respectively [10]. These data clearly exhibit precisely the speed-accuracy trade-off. As mentioned in the previous section, changes in 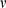 may potentially capture this effect. Indeed, decreasing the input common to both populations increases reaction-times and performance, while increasing the input has the opposite effects. As seen in Figure 4, changes in the input common to both populations, i.e. 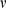 do in fact capture the speed-accuracy trade-off.

Since Equation 8 can be derived analytically from more complex model systems, we can map the values of the coefficients obtained from fits to behavioral data back to more physiologically meaningful parameters. An example of this is shown in Figures 3 and 4 (red symbols) where we have conducted simulations of the rate equations Equations 11–13 with parameter values chosen to match the coefficients from the fit, using Equations 14–17. Thus one can trivially fit higher dimensional models to data once Equation 8 has been derived.

The fits of the nonlinear diffusion equation Equation 8 to behavioral data suggest not only that the putative decision making circuit behaves in a way consistent with a winner-take-all framework, but that changes in inputs to this circuit alone are sufficient to account for performance and mean reaction-times. In addition, the best fits to the data were found for |ηΔν|, 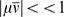, i.e. in the vicinity of the bifurcation. This provides an a posteriori validation of the use of Equation 8 to describe these data since it represents, after all, a reduction of the dynamics near the bifurcation. Moreover, it is precisely in this regime that the dynamics of Equation 8 most closely resembles that of the linear diffusion equation.

## Discussion

One-dimensional diffusion equations have long been used to model behavior in two-choice reaction-time tasks. Recently, researchers discovered that the trial-averaged single-unit activity recorded in areas of the brain which are implicated in generating this behavior closely resemble the dynamics of a linear diffusion process [21]. This suggests a correspondence between the neural activity in these areas and the decision making variable *X* in the linear diffusion equation. However, it remained unclear how the cortical activity might actually conspire to generate such a linear diffusion. On the other hand, it was soon demonstrated that some aspects of the neural activity could be captured in biophysically motivated winner-take-all network models [28]–[30]. Here we have shown, through the use of standard tools from nonlinear dynamics theory, that the dynamics in winner-take-all models relevant for two-choice decision making can be captured in a one-dimensional nonlinear diffusion equation, Equation 8. This suggests that the cortical circuits involved in decision making generically generate an effective nonlinear diffusion which in a limited parameter regime leads to behavior very similar to that predicted by the linear diffusion equation.

The dependence of the coefficients in Equation 8 on external inputs is explicit and independent of the details of the underlying model. This suggests that the functional dependence of behavioral measures in two-choice decision making on changes in inputs is universal. In particular, we predict that modulations of the input common to both populations can account for the speed-accuracy trade-off. This mechanism differs from that evoked by others previously, which consists of varying the threshold for detection of the decision (e.g. a higher threshold increases reaction times and increases performance), [6],[30]. The novel mechanism proposed here of speed-accuracy trade-off through modulations in the mean input predicts that pre-stimulus activity in LIP should be higher, on average, when the subject must respond more rapidly. Support for this comes from the observation that the baseline neuronal activity in monkeys varies in a task-dependent manner, see Figure 16 from [20], a phenomenon which has been interpreted as anticipatory activity. Indeed, increases in the baseline activity were found to correlate with more rapidly evolving post-stimulus activity. Equation 8 now provides us with an explanation for the functional role of this activity. This phenomenon could be further confirmed through comparison of the relative changes in the BOLD signal in fMRI studies of activity in brain areas in humans homologous to LIP during the pre-stimulus period in a task where the speed-accuracy trade-off is observed behaviorally.

While Equation 8 appears similar in form to other diffusion models which have been used to describe behavior in two-choice decision making [9], [12]–[14],[45], it is important to distinguish between their very distinct mathematical pedigrees. In particular, we have not evoked the nonlinear diffusion equation as a phenomenological model of behavior for two-choice decision making. Rather, it represents the correct asymptotic description of the dynamics in nonlinear winner-take-all models near the bifurcation to winner-take-all behavior. This observation has two consequences. Firstly, in as far as nonlinear winner-take-all models can successfully reproduce some qualitative features of the neuronal activity in brain areas implicated in the decision making process for two-choice decision making [28], i.e. LIP, the nonlinear diffusion equation also provides an approximate description of this activity. Secondly, if an actual nonlinear winner-take-all process is at work in the brain during such tasks, then this process will behave as an approximately one-dimensional diffusion process in the vicinity of the bifurcation to winner-take-all behavior. This process is described by the nonlinear diffusion equation Equation 8. Note also that the effective reduction in dimension of the dynamics in nonlinear systems in general only occurs at bifurcations. Thus nonlinear normal forms for bifurcations such as Equation 8 represent the only proper one-dimensional reduction of such a system.

As in the linear diffusion equations, bias in external inputs in the nonlinear diffusion equation appears to leading order as a constant drift term. In contrast, while reductions of linear connectionist models to the linear diffusion equation lead to a linear (Ornstein-Uhlenbeck) term proportional to the difference between intrinsic ‘leak’ and the effective cross inhibition, this is not the case in nonlinear systems. Rather, this term reflects the linear growth rate of the spontaneous state which, given that the input is the bifurcation parameter, is simply proportional to the distance of the common external input from the critical value at the bifurcation. Thus this term varies with modulations of the external input, unlike in the linear case. Finally, the cubic nonlinearity, which is the lowest order nonlinearity consistent with the reflection symmetry of the original system, leads to an inverted-U potential. This drives the activity to infinity in finite time, reflecting the escape from the spontaneous state to the ‘decision’ state. As illustrated in Figure 1, this renders the measurement of reaction-times and performance insensitive to the exact placement of a threshold as long as it is high enough. Setting relatively high thresholds therefore effectively eliminates one free parameter from the model, namely the threshold placement. Nonetheless, one could set low thresholds in the nonlinear system, i.e. very close to the spontaneous state [30]. It has been hypothesized that the threshold for detection of a decision in the brain may be set by downstream areas including superior colliculus [30] or the basal ganglia [46]

As it turns out, Equation 8 can account for behavioral data for the random moving dot task in monkeys and humans, c.f. Figures 3 and 4. As such Equation 8 seems to provide a correct description of both the neuronal activity and the behavior in this task, thereby linking the two. This, however, in no way contradicts the success of connectionist and linear diffusion models in fitting behavioral data. Indeed, a comparison of the nonlinear diffusion equation and the linear one, Equation 1, shows approximately equally good fits for correct reaction-times and performance for the data in Figures 3 and 4, see supporting material (Text S1). On the other hand error reaction-times in Figure 3, which are longer than correct ones, cannot be fit by the linear diffusion model unless variability in the initial condition and drift term across trials is introduced [9]. They are, however, correctly captured by the nonlinear diffusion equation. We note, furthermore, that several groups have derived reduced models for two-choice decision making. Wong and Wang performed a heuristic reduction of a spiking network model to a system of two coupled rate equations [29], and showed that it gave similar qualitative behavior. As a canonical model, Equation 8 qualitatively captures the dynamics of both the network and the rate models, also see fit in supporting material (Text S1). We note, however, that far from the bifurcation the full dimensionality of the system being studied will come into play and the dynamics will not be captured by Equation 8. Much of the phenomenology in [29] appears to occur in this regime. Once this is the case, the dynamics may depend crucially on the details and dimensionality of the system and, if so, cannot be generalized. Wong et al. have recently used their reduced model to explain the experimentally observed violation of time-shift invariance in the behavior of monkeys doing the random moving dot task [47], lending further support for the nonlinear, attractor network framework for LIP activity [31]. They also note that the inclusion of target inputs, which more faithfully reproduces the experimental paradigm, ‘renders the model behavior closer to a one-dimensional model in the decision process’ [31]. Interestingly, the presence of the unstable cubic term in the 1D nonlinear diffusion equation Equation 8 should lead to the experimentally observed violation of time-shift invariance for which perturbations arriving later in time have a lesser effect due to the nonlinear acceleration away from the spontaneous state. This remains to be tested quantitatively. Usher and McClelland derived a one-dimensional diffusion equation equivalent to an Ornstein-Uhlenbeck process from a neurobiologically motivated system of two coupled, threshold-linear equations [12]. This and other similar systems of linear equations were studied by Bogacz, Brown and collaborators [13],[14]. The linearity of the system in these studies allowed for an in-depth analytical characterization of the dynamics. Indeed, it has been argued that neurobiologically motivated models might, within certain parameter regimes, be reducible to an equivalent linear diffusion equation [14]. However, as we have shown here, if the underlying winner-take-all system exhibits any generic nonlinearities, as seems to be the case in neural systems, the correct dynamics are given by Equation 8.

Soltani and Wang [48] and Fusi et al. [49] have both investigated how synaptic plasticity might shape the response in winner-take-all decision making circuits. Soltani and Wang introduced a reward-dependent stochastic Hebbian rule for updated synaptic strengths which successfully reproduces the so-called ‘matching behavior’ while Fusi et al. have presented a model of flexible sensorimotor mapping in which reward-dependent synaptic plasticity shapes the output of a winner-take-all decision making circuit. In both cases, the performance depends on the difference in the fraction of potentiated synapses between the two populations Δ*c*, i.e. the symmetry breaking occurs due to plastic changes in synaptic strength. In the context of Equation 8 this would lead to an additional term 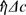 which is functionally equivalent to the symmetry-breaking term proportional to the difference in inputs. The effect of synaptic plasticity in two-choice decision making could therefore be studied by means of Equation 8 coupled with an appropriate learning rule.

The reduction to Equation 8 is strictly valid only in the immediate vicinity of the bifurcation. For this reason it might be argued that the current scenario is tantamount to fine-tuning and may not be biologically relevant. Three facts indicate this is not the case. (I) As we have shown here Equation 8 can be rigorously derived from model systems and can provide a *quantitative* match even away from the bifurcation. (II) Equation 8 can be fit to behavioral data, previously published model networks [28] and models in regimes far from the bifurcation where a quantitative match is no longer found. It thus provides a correct qualitative description of the dynamics. Furthermore these fits are made by varying physiologically meaningful parameters in ways that are either consistent with experimental findings or which lead to experimentally testable predictions. (III) Lastly, a large literature exists showing that human behavior in 2-choice decision making is well-described by one-dimensional sequential sampling models. A deep question is how such low-dimensional dynamics might arise from high-dimensional neuronal dynamics. We believe the most parsimonious explanation is that the neuronal circuits involved operate near the low dimensional manifold which arises naturally within a certain parameter range, i.e. near the bifurcation.

## Materials and Methods

### Derivation of the Nonlinear Diffusion Equation from Equations 11–13

Here we derive the nonlinear diffusion equation (noise driven amplitude equation for an imperfect pitchfork bifurcation) from Equations 11–13. We first study the linear stability of the spontaneous fixed point, analogously to Equation 6 and then extend this analysis to take into account nonlinear effects in a so-called *weakly nonlinear* analysis using a multiple-scales approach.

### Linear Stability

We assume that *I_A_* = *I_B_* = *I* and consider an ansatz of the form (*r_A_*, *r_B_*, *r_I_*) = (*R*,*R*,*R_I_*)+(Δ*r_A_*,δ*r_B_*,δ*r_I_*)*e* ^λ*t*^, where *R* = Φ(*sR*−*cR_I_*+*I*) and *R_I_* = Φ*_I_*(2*gR*+*I_I_*). This leads to an eigenvalue problem of the form
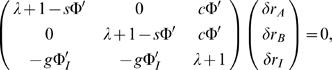
(18)where the derivatives of the transfer functions are evaluated at the fixed point.

The eigenvalue corresponding to the eigenvector (1,−1,0) is equal to zero for 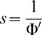.

### Weakly Nonlinear Dynamics

We expand the input current and the rates around the steady instability found above. We take
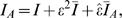
(19)

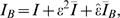
(20)

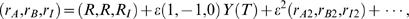
(21)where ε and 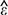 are small parameters which measure the distance from the bifurcation and the difference in inputs to the two excitatory populations respectively. Near the bifurcation, the mode corresponding to the critical eigenvector *Y*(*T*) evolves on the slow time scale *T* = ε^2^ *t*. The expansions given above are plugged into Equations 11–13 and terms are collected order by order. We assume that 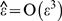, i.e. weak symmetry breaking. The scaling of input currents in ε is dictated by the reflection symmetry of the original system, i.e. we expect a pitchfork bifurcation. Were we to not use the knowledge of this symmetry, a more general expansion of the currents, including all orders of ε could be used, leading to the same result. That is, we would find for example that the term proportional to ε is identically equal to zero, etc.

#### O(ε)

We recover the linear stability problem
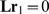
(22)where
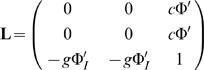
(23)and **r**_1_ = (1,−1,0)*Y*(*T*).

#### O(ε^2^)



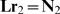
(24)

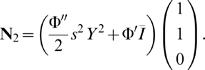
(25)The matrix **L** cannot be inverted to solve for **r**_2_. A solution therefore only exists if the vector **N**_2_ is in the left-null eigenspace of the linear operator. This can be expressed as 〈**r** ^†^,**N_2_**〉 = 0 where **r** ^†^ = (1,−1,0) and the *inner product* 〈**x**,**y**〉 is here equivalent to the dot product **x** *^T^*·**y**. This condition is met upon inspection. The solution **r**_2_ can then be found by projecting onto the eigenspace orthogonal to the left-null eigenvector, i.e. 〈(1,1,1),**Lr**_2_−**N**_2_〉 = 0 and 〈(1,1,−2),**Lr**_2_−**N**_2_〉 = 0. Doing so yields
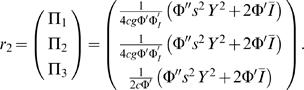
(26)


#### O(ε^3^)

We have
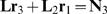
(27)where
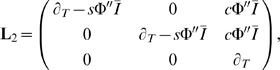
(28)

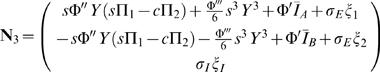
(29)Again, Equation 27 only has a solution if 〈**r** ^†^,**L**_2_ **r**_1_−**N**_3_〉 = 0. This leads to the equation
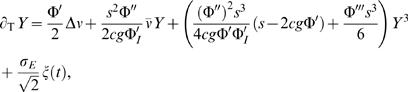
(30)where Δν = *I_A_*−*I_B_* and 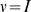. By rescaling the amplitude as *Y* = |γ|^1/2^ *X*, we arrive at Equation 8 with coefficients given by Equations 14–17.

### Fits from Nonlinear Diffusion Equation

We solve for the performance and reaction time in Equation 8 (solid lines in Figures 3 and 4) by numerically evaluating *RT*(0) and *P*(0), Equations S2 and S4, using Romberg integration [50], with limits of integration of ±0.21 and ±0.19 for Figures 3 and 4 respectively. The value of *RT* and *P* are relatively insensitive to increases in the limits of integration, related to the fact that in Equation 8, *X* approaches ±∞ in finite time. We have also fit the data from Figures 3 and 4 using direct numerical simulation of Equation 8 with a threshold of ±1, obtaining results for *RT* which vary by no more than a constant shift of 10 ms. Fits in Figures 3 and 4 are made by eye.

### Parameters for Rate Equations: Equations 11–13

Once the fits have been made using the nonlinear diffusion equation, we must choose parameters in the rate equations which give the proper values for the coefficients, using the expressions Equations 14–17. Various parameter combinations are possible, indicative of the reduction in dimensionality of the system and a potential mechanism for robustness in functionality.

For the simulations in Figure 3 we took 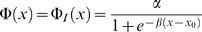 with α = 1.5, β = 2.5 and *x*_0_ = 1, *s* = 1.9, *c* = 1, *g* = 1, *I_I_* = 0.2, *I* = 0.3695, σ_E_ = σ*_I_* = 0.001634 *I*_1_−*I*_2_ = 2.168*e*−05×*coherence*.

For the simulations for subject 1 in Figure 4 we took 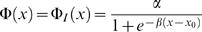 with α = 1.5, β = 2.5 and *x*_0_ = 1, *s* = 1.9, *c* = 1, *g* = 1, *I_I_* = 0.2, *I* = 0.3675,0.3687,0.3742, σ_E_ = σ*_I_* = 0.001634 *I*_1_−*I*_2_ = 4.066*e*−05×*coherence*.

For the simulations for subject 2 in Figure 4 we took 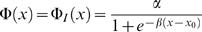 with α = 1.5, β = 2.5 and *x*_0_ = 1, *s* = 1.9, *c* = 1, *g* = 1, *I_I_* = 0.2, *I* = 0.3673,0.3684,0.3721, σ_E_ = σ*_I_* = 0.001634 *I*_1_−*I*_2_ = 4.228*e*−05×*coherence*.

In all cases, a trial ends once one of the rates crosses a fixed threshold of 0.7. Initial condition was *r_A_* = *r_B_* = 0.16, *r_I_* = 0.35 where the values at the bifurcation are *r_A_* = *r_B_* = 0.253 and *r_I_* = 0.486. Changing the initial condition did not alter the results significantly (not shown). We conducted 10,000 runs for each value of the coherence.

## Supporting Information

Text S1 Detailed description of nonlinear diffusion equation: closed-form expressions for RT and P and derivations from three model systems. Comparison between nonlinear and linear diffusion models.
(0.51 MB PDF)
