# Supplementary material for: Neurobiological Models of Two-Choice Decision Making Can Be Reduced to a One-Dimensional Nonlinear Diffusion Equation
Source: PLoS Comput Biol. 2008 Mar 28;4(3):e1000046. doi: 10.1371/journal.pcbi.1000046 (PMC2268007; doi:10.1371/journal.pcbi.1000046)
Supplement: Text S1 — Detailed description of nonlinear diffusion equation: closed-form expressions for RT and P and derivations from three model systems. Comparison between nonlinear and linear diffusion models. (0.51 MB PDF) [file pcbi.1000046.s001.pdf]

Contents

In section 1 we discuss the derivation, validity and some dynamical properties of the nonlinear diffusion equation. This includes:

- Section 1.1: When does the equation break down?
- Section 1.2: Higher order terms in the nonlinear diffusion equation.
- Section 1.3: Performance and Reaction-time for the nonlinear diffusion equation.
- Section 1.4: Comparisons of fits from the linear and the nonlinear diffusion equations.

In section 2 we derive the nonlinear diffusion equation from two additional model systems beyond the system of three rate equations studied in the main text. The sections are:

- Section 2.1: Network of integrate-and-fire neurons.
- Section 2.2: Reduced equations from Wong and Wang 2006.

1 The Nonlinear Diffusion Equation

Canonical equations for bifurcations such as the nonlinear diffusion equation are known as amplitude equations or normal forms. Here we briefly discuss the validity of the amplitude equation approach for systems with winner-take-all behavior. For a general introduction to bifurcations see [8] and [3] while a more in-depth treatment can be found in [5], [4], and [10].

We first note that a bifurcation is a *qualitative* change in the dynamics of a system as a function of a system parameter. In the context of neurobiologically motivated winner-take-all models the parameter of interest is the input to the system which is modulated by the absence or presence of the stimulus amongst other possible sources. The qualitative change in behavior of interest is from the presence of a single stable state, i.e. spontaneous activity, to a state in which the dynamics can appropriately be labeled 'winner-take-all'.

We first consider a network capable of winner-take-all behavior in which the populations of neurons, A and B, which encode the two possible choices

are statistically identical, i.e. no characteristic stands out which would allow us to tell neurons from the two populations apart a priori and any heterogeneities in single-cell properties or network topology are shared by both populations alike. If both populations receive the same input, e.g. a two-choice random dots task with zero coherence, then the system behavior remains unchanged if we interchange the identity of the two populations $(A, B) \rightarrow (B, A)$. Because of this reflection symmetry, if there is a steady (non-oscillatory) bifurcation of the spontaneous state to a winner-take-all state, it will have the form $\dot{X} = aX \pm X^3$, i.e. it is a pitchfork bifurcation, maintaining the reflection symmetry $X \rightarrow -X$.

If, as is our case, the bifurcation parameter is the input, then the linear growth rate $a = \mu\bar{\nu} = \mu(\nu - \nu_{bif})$ where ν is the mean input to both populations, and ν_{bif} is the input right at the bifurcation. Now if the reflection symmetry is broken by a small difference in input $\Delta\nu$ to the two populations, this will generically add a constant term, proportional to that difference. If we additionally assume the presence of a noise term we have

$$\dot{X} = \eta\Delta\nu + \mu\bar{\nu}X \pm X^3 + \sigma\xi(t), \tag{S.1}$$

where σ is the noise amplitude and we have assumed additive noise.

Note that while we can not know the functional form of η, μ and σ without starting with a model and actually deriving Eq(S.1), we find that the constant and linear terms are proportional to the difference in inputs and the mean input to both populations respectively. This result is therefore independent of our choice of model.

1.1 When does the equation break down?

Eq.(S.1) holds in a strict mathematical sense when we are vanishingly close to the bifurcation and the difference in inputs is infinitesimally small. In any real application it will give a quantitative description of the dynamics in some limited range of parameter values which is model-dependent. Fortunately, amplitude equations such as Eq.(S.1) tend to capture the qualitative features of the dynamics even relatively far from the bifurcation. Although the only way to test the region of *quantitative* validity of Eq(S.1) for a given model is to compare with the said model, we can discuss the conditions under which quantitative predictions should deteriorate.

1. As the bifurcation parameter, in this case the external input ν, deviates from the value at the bifurcation, any quantitative predictions will worsen.

2. As the symmetry-breaking term, in this case the difference in external inputs, increases, any quantitative predictions will worsen.

3. As the amplitude itself increases, i.e. the value of X, within a simulation, higher order terms, not present in Eq(S.1) will gain in importance and the quantitative match will worsen.

4. Since we are not interested in stationary behavior, i.e. fixed points or limit cycles, but rather transient phenomenon, we must additionally be concerned about initial conditions and noise. Close to the bifurcation, we can approximate the dynamics starting from an initial condition. The effect of initial conditions in a particular system has, in fact, been described in detail in [11] where it was argued that the initial transient during which the system relaxes to the slow manifold cannot be ignored. However, sufficiently close to the bifurcation this relaxation is rapid and merely introduces an error into the quantitative predictive power of Eq.(S.1). We treat the noise as a small-amplitude forcing with the consequence that the noise term for the amplitude equation can be obtained through a simple linear transformation. The accuracy of this approach degrades continuously with increasing noise amplitude.

   In fact, even where the quantitative error is large or where no such quantitative comparison can be made, Eq(S.1) provides an excellent qualitative description of the relevant behavior measures. An example can be seen in Fig.S.1 where we have fit Eq(S.1) to data from the spiking model in [9]. Although the complexity of the model does not allow for an analytical derivation of Eq.S.1, the equation nonetheless provides an excellent fit to data generated from numerical simulation of the network.

1.2 Higher order terms in the nonlinear diffusion equation

Eq.S.1 represents the correct asymptotic description of the noisy dynamics near an imperfect pitchfork bifurcation in general. However, under some

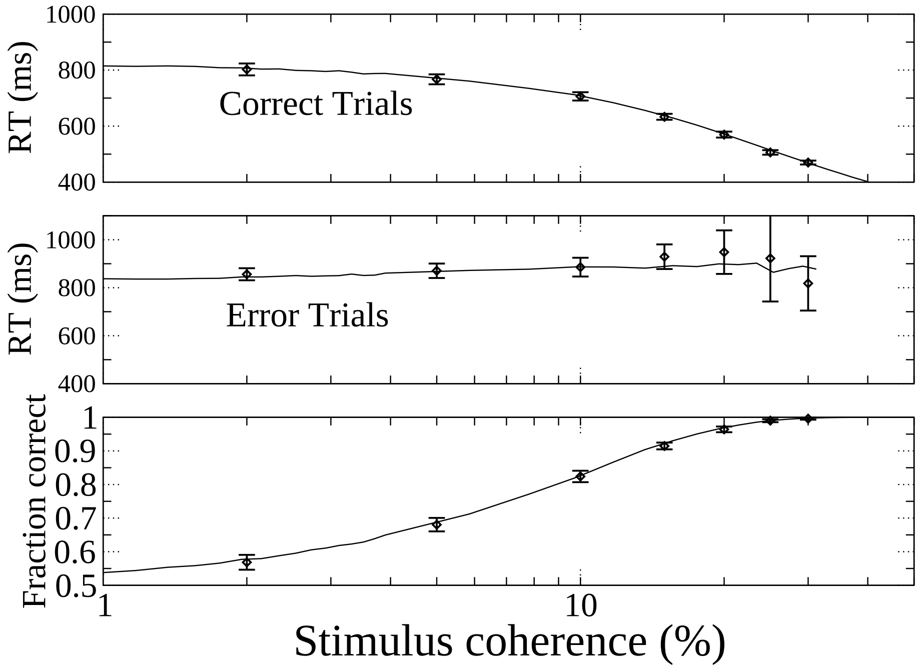

Fig. S.1: The nonlinear diffusion equation gives a good fit to simulations from network models, even when it cannot be analytically derived. Here we compare results from Eq.S.1 (solid lines) with data generated from simulation of a network model including synapses of the AMPA, GABA and NMDA type [9]. The presence of these currents makes an analytical derivation of Eq.S.1 intractable. The network model and parameters values are as in [9]. Parameters for Eq.S.1 were $\mu\bar{\nu} = 0.003$, $\sigma = 0.000676$ and $\eta\Delta\nu = \text{coherence}/3.9\text{e} - 5$.

circumstances higher order terms (strictly asymptotically smaller than those in Eq.S.1) may gain in importance. We consider two cases here briefly. First we note that the unnormalized version of Eq.S.1 can be written $\dot{X} = \eta + \mu X + \gamma X^3 + \sigma\xi(t)$.

1. Near the point in parameter space where the cubic term changes sign, i.e. $|\gamma| \ll 1$, all terms should be rescaled to include a saturating quintic term $-|\Gamma|X^5$. Unless the cubic term is very small this rescaling is not mathematically justifiable and the quintic term should not be included.

2. Multiplicative noise: If the noise source in the original equation is multiplicative in nature then the leading order noise term in the amplitude equation is essentially given by the same noise source rescaled by the steady-state solution and is therefore additive. A higher order term involves the interaction between the noise and the small-amplitude solution X of Eq.S.1 and is therefore a correction of the linear term μ.

1.3 Performance and reaction time for the non-linear diffusion equation

In this section we will describe how the performance and reaction time depends on some of the parameters of the non-linear diffusion equation.

For simplicity we redefine the following parameters in Eq.S.1

$$\alpha = \eta\Delta\nu, \quad \text{and } \beta = \mu\bar{\nu}.$$

It is convenient to introduce the function $Q(X) = \frac{2}{\sigma^2}E(X)$, where $E(X)$ is Eq.10 from the main text and following Karlin & Taylor (1981) we will use the so-called scale function $S(y)$ and so-called speed density $m(y)$ defined by

$$S(y) = \int_{-a}^{y} \exp\{Q(z)\}dz, \quad m(y) = \frac{1}{\sigma^2 \exp\{Q(y)\}}.$$

Given two symmetric boundaries a and $-a$ ($a > 0$) the probability of the process exiting through a, as a function of the initial condition X_0, $-a < X_0 < a$, is given by [6, p.195]

$$P_a(X_0) = \frac{\int_{-a}^{X_0} \exp\{Q(y)\}dy}{\int_{-a}^{a} \exp\{Q(y)\}dy} = \frac{S(X_0)}{S(a)}. \tag{S.2}$$

The probability of exiting through the other boundary (i.e. through $-a$) is given by $1 - P_a$.

1.3.1 Expressions for the mean reaction time

The mean first passage time from the interval $(-a, a)$, as a function of the initial condition X_0 is given by ([6, p.197])

$$\begin{aligned} RT(X_0) =& 2P_a(X_0) \int_{X_0}^{a} [S(a) - S(y)]m(y)dy + \\ & 2(1 - P_a(X_0)) \int_{-a}^{X_0} [S(y) - S(-a)]m(y)dy. \end{aligned} \tag{S.3}$$

This expression (Eq. S.3) gives the mean exit time from $(-a, a)$ through any of the boundaries. However, often it is of interest to consider the mean exit time through a particular boundary. For example, we might want to know the mean exit time through a starting somewhere in $(-a, a)$, as this could correspond to a correct decision. To find this we need to consider the conditional diffusion process consisting of all sample paths of the original diffusion process that eventually exit through a. The expression then reads (see [6, p.264])

$$\begin{aligned} RT_a(X_0) =& \frac{2[S(a) - S(X_0)]}{S(a)S(X_0)} \int_{-a}^{X_0} S^2(y)m(y)dy + \\ & 2\int_{X_0}^{a} \frac{S(y)[S(a) - S(y)]}{S(a)} m(y)dy. \end{aligned} \tag{S.4}$$

1.3.2 How performance depends on the parameters of the non-linear diffusion equation

To study how the performance function (Eq. S.2) depends on changes of the model parameters we here investigate the derivative of the performance function with respect to these parameters. First we derive a general expression for the derivative with respect to a generic parameter γ:

$$\frac{\partial (P_a(X_0))}{\partial \gamma} = \frac{1}{\int_a^b \exp\{Q(y)\}dy} \left[\frac{\partial (\int_a^{X_0} \exp\{Q(y)\}dy)}{\partial \gamma} - P_a(X_0) \frac{\partial (\int_a^b \exp\{Q(y)\}dy)}{\partial \gamma} \right].$$

Next we use Leibniz's rule to take the derivative under the integral sign to get:

$$\frac{\partial(P_a(X_0))}{\partial\gamma} = \frac{1}{\int_{-a}^{a}\exp\{Q(y)\}dy}\left[\int_{-a}^{X_0}\frac{\partial(Q(y))}{\partial\gamma}\exp\{Q(y)\}dy - P_a(X_0)\int_{-a}^{a}\frac{\partial(Q(y))}{\partial\gamma}\exp\{Q(y)\}dy\right]. \tag{S.5}$$

1.3.3 Dependence on α

We first consider the dependence on α (remember that $\alpha = \eta\Delta\nu$). To proceed we use the following observations

$$\frac{\partial(Q(y))}{\partial\alpha} = \frac{-2y}{\sigma^2},$$

and

$$\int_{-a}^{a}\exp\{Q(y)\}dy > 0$$

for any values of the parameters. Then, after some rearrangements, we get that

$$\frac{\partial(P_a(X_0))}{\partial\alpha} = c\left[P_a(X_0)\int_{X_0}^{a} y\exp\{Q(y)\}dy - (1 - P_a(X_0))\int_{-a}^{X_0} y\exp\{Q(y)\}dy\right],$$

where $c = 2(\sigma^2\int_{-a}^{a}\exp\{Q(y)\}dy)^{-1} > 0$ and does not depend on the initial condition. By changing the variable of integration in the last integral to $y = -y$ we get

$$\frac{\partial(P_a(X_0))}{\partial\alpha} = c\left[P_a(X_0)\int_{X_0}^{a} y\exp\{Q(y)\}dy + (1 - P_a(X_0))\int_{-X_0}^{a} y\exp\{Q(-y)\}dy\right].$$

Since $a > 0$, by assumption, the first integral in the above expression is clearly positive. The second integral can be rewritten as

$$\int_{-X_0}^{a} y\exp\{Q(-y)\}dy = \int_{0}^{X_0} y\left(\exp\{Q(-y)\} - \exp\{Q(y)\}\right)dy + \int_{X_0}^{a} y\exp\{Q(-y)\}dy,$$

which shows that it also is positive. Hence it follows that

$$\frac{\partial(p_a(X_0))}{\partial\alpha} > 0.$$

That is to say, performance is an increasing function of α.

1.3.4 Dependence on β

Next we will show that P_a is a decreasing function of β as long as $P_a > 0.5$ (remember, $\beta = \mu\bar{\nu}$). Using that

$$\frac{\partial(Q(y))}{\partial\beta} = \frac{-y^2}{\sigma^2},$$

and proceeding as above we arrive at:

$$\frac{\partial(P_a(X_0))}{\partial\beta} = d\left[P_a(X_0)\int_{X_0}^{a} y^2\exp\{Q(y)\}dy - (1-P_a(X_0))\int_{-X_0}^{a} y^2\exp\{Q(-y)\}dy\right],$$

where $d = (\sigma^2\int_{-a}^{a}\exp\{Q(y)\}dy)^{-1} > 0$ and does not depend on the initial condition. We can, without loss of generality, restrict our considerations to the case of $P_a(X_0) > 0.5$.[1] Doing this it is clear that the only way that $\frac{\partial(P_a(X_0))}{\partial\beta}$ can be negative is if the second integral is bigger than the first. This means that if we can show that the performance is a decreasing function of β for the initial condition $X_0 = 0$ it will also follow that this is true for all initial conditions $X_0 \geq 0$. This is so because a positive initial condition will enhance the differences between the two integrals. So assume that $X_0 = 0$.

Then to prove that the derivative is always negative amounts to showing that

$$\frac{1}{1-P_a(X_0)}\int_0^a y^2\exp\{Q(y)\}dy < \frac{1}{P_a(X_0)}\int_0^a y^2\exp\{Q(-y)\}dy. \qquad \text{(S.6)}$$

To do this we introduce the two functions

$$g(y) = \frac{\exp\{Q(y)\}}{\int_{-a}^{a}\exp\{Q(z)\}dz(1-P_a(X_0))}, \quad h(y) = \frac{\exp\{Q(-y)\}}{\int_{-a}^{a}\exp\{Q(-z)\}dz(P_a(X_0))}.$$

[1] This is so because if we can show that in this case P_a is a decreasing function of β it follows that P_{-a} is an increasing function.

Note that since

$$\int_{-a}^{a} \exp\{Q(-z)\}dz = -\int_{a}^{-a} \exp\{Q(z)\}dz = \int_{-a}^{a} \exp\{Q(z)\}dz,$$

showing that (S.6) holds amounts to showing that

$$\int_0^a y^2(g(y) - h(y))dy < 0. \tag{S.7}$$

Next we note that $\int_0^a g(y)dy = 1$ and $\int_0^a h(y)dy = 1$. This implies that unless g and h are identical (i.e. if $\alpha = 0$) there must be intervals where $g > h$ and vice-verse. Given the form of h and g it is easy to show that if $\alpha \neq 0$ there is exactly one point $y^* > 0$ at which $g(y^*) = h(y^*)$. Moreover, for any y in the half-open interval $[0, y^*)$ we have that $g(y) > h(y)$, similarly for $y \in (y^*, a]$ we have that $g(y) < h(y)$. This means that we can decompose the integral in (S.7) as follows

$$\int_0^a y^2(g(y) - h(y))dy = \int_0^{y^*} y^2(g(y) - h(y))dy - \int_{y^*}^a y^2(h(y) - g(y))dy.$$

Now the integrands in the two integrals on the right hand side are both positive and we will proceed by giving an upper bound of the first and a lower bound of the second. Indeed we have that

$$\begin{aligned}\int_0^a y^2(g(y) - h(y))dy =& \int_0^{y^*} y^2(g(y) - h(y))dy - \int_{y^*}^a y^2(h(y) - g(y))dy \leq \\ & y^{*2}\int_0^{y^*} (g(y) - h(y))dy - y^{*2}\int_{y^*}^a (h(y) - g(y))dy = \\ & y^{*2}\int_0^a g(y) - h(y)dy = 0,\end{aligned}$$

and hence that performance is a non-increasing function of β. Note that this statement is valid for non-negative initial conditions and only in the case when $p_a > 0.5$.

1.3.5 How reaction-time depends on β

Next we demonstrate that the mean RT is a decreasing function of β. We do this for the case of $\alpha = 0$ and initial condition $X_0 = 0$. This implies that

$P_a = P_{-a} = 0.5$ and that $Q(y) = Q(-y)$ and $m(y) = m(-y)$ in which case Eq. S.3 becomes

$$RT(X_0 = 0) = 2 \int_0^a [S(a) - S(y)] m(y) dy.$$

Next, we use that

$$\frac{\partial(S(a) - S(y))}{\partial \beta} = -\frac{1}{\sigma^2} \int_y^a y^2 \exp\{Q(y)\} dy,$$

and that

$$\frac{\partial(m(y))}{\partial \beta} = \frac{1}{\sigma^2} y^2 m(y).$$

Applying to $t(X_0)$ gives

$$\begin{aligned}\frac{\partial(RT(X_0 = 0))}{\partial \beta} &= -\frac{1}{\sigma^2} \int_0^a \left(\int_y^a z^2 \exp\{Q(z)\} dz \right) m(y) dy \\ &+ \frac{1}{\sigma^2} \int_0^a \left(\int_y^a \exp\{Q(z)\} dz \right) y^2 m(y) dy.\end{aligned}$$

Hence $\partial(RT(X_0))/\partial \beta < 0$ if the first term is bigger than the second. But this must be so since

$$\int_y^a z^2 \exp\{Q(z)\} dz \geq y^2 \int_y^a \exp\{Q(z)\} dz$$

for all $y \geq 0$, and we are done.

1.4 Comparisons of fits from the linear (constant drift) and nonlinear diffusion equations.

Here we compare fits from the linear and nonlinear diffusion equations for data from the random moving dot task, see also Figs.3 and 4 from the main text. We use here the 'standard' diffusion model without variability in either the initial condition or drift across trials. Curves for the data from [7] were reproduced using standard closed form expressions for performance and reaction-times and parameter values given in [7]. The fits of both the linear and nonlinear diffusion models were made by minimizing the sum of the normalized squared difference between data and model(s). We only included

mean RT on correct trials and percent correct in the fitting. The normalization was done by dividing the difference of two particular quantities by their sum. This was to give approximately the same weight to all observations. For the linear model the minimization was made by the quasi-Newton method `bfgsmin` as implemented in the open source software package `octave`. Minimizing the nonlinear model requires exquisite control over the step-size and was made by an semi-automatic gradient-based algorithm. Evaluating the integrals for performance and reaction-time stated above numerically is computationally expensive and made a more exhaustive search unfeasible. Therefore the minima obtained for the nonlinear model might not be global. In fitting the models to the data from [7] we constrained the fits and only allowed the threshold and residual RTs to vary between conditions in the linear model and linear term and residual RTs in the nonlinear one. Parameter values and errors for the fits are shown in the tables below.

linear model	k	A	res. RT	fitting error
Monkeys	13.5	0.70	327.2	8.17e-5
Subj1 05	22.0	0.45	305.0	3.22e-4
Subj1 10	22.0	0.86	320.5	4.07e-4
Subj1 20	22.0	1.28	284.0	3.48e-3
Subj2 05	26.8	0.49	300.9	2.17e-4
Subj2 10	26.8	0.88	304.4	9.43e-4
Subj2 20	26.8	1.38	269.7	1.23e-3

Here k and A are the normalized drift and threshold, see [7] for details.

nonlinear model	σ	η	μ	res. RT	fitting error
Monkeys	1.35e-3	6.67e-6	3.0e-3	230	6.5e-5
Subj1 05	1.15e-3	9.35e-6	1.03e-2	219.5	6.1e-4
Subj1 10	1.15e-3	9.35e-6	1.60e-3	215.5	5.3e-4
Subj1 20	1.15e-3	9.35e-6	-3.96e-4	212.5	2.6e-3
Subj2 05	1.37e-3	1.30e-5	8.00e-3	213.5	5.4e-4
Subj2 10	1.15e-3	1.30e-5	1.08e-2	213.5	1.4e-3
Subj2 20	1.15e-3	1.30e-5	-1.14e-3	221.5	1.3e-3

The fits to the data can be seen in Fig.S.2.

The nonlinear model performed better than the linear model (in the sense of having a smaller error) in fitting the data from Roitman and Shadlen and in one of the subjects (subject 2) in the data from Palmer et al. The linear model performed better on the other subject of the Palmer et al. data. Note that the linear model predict that error reaction times are the same as the correct reaction times which for the Roitman and Shadlen data is not the case. This

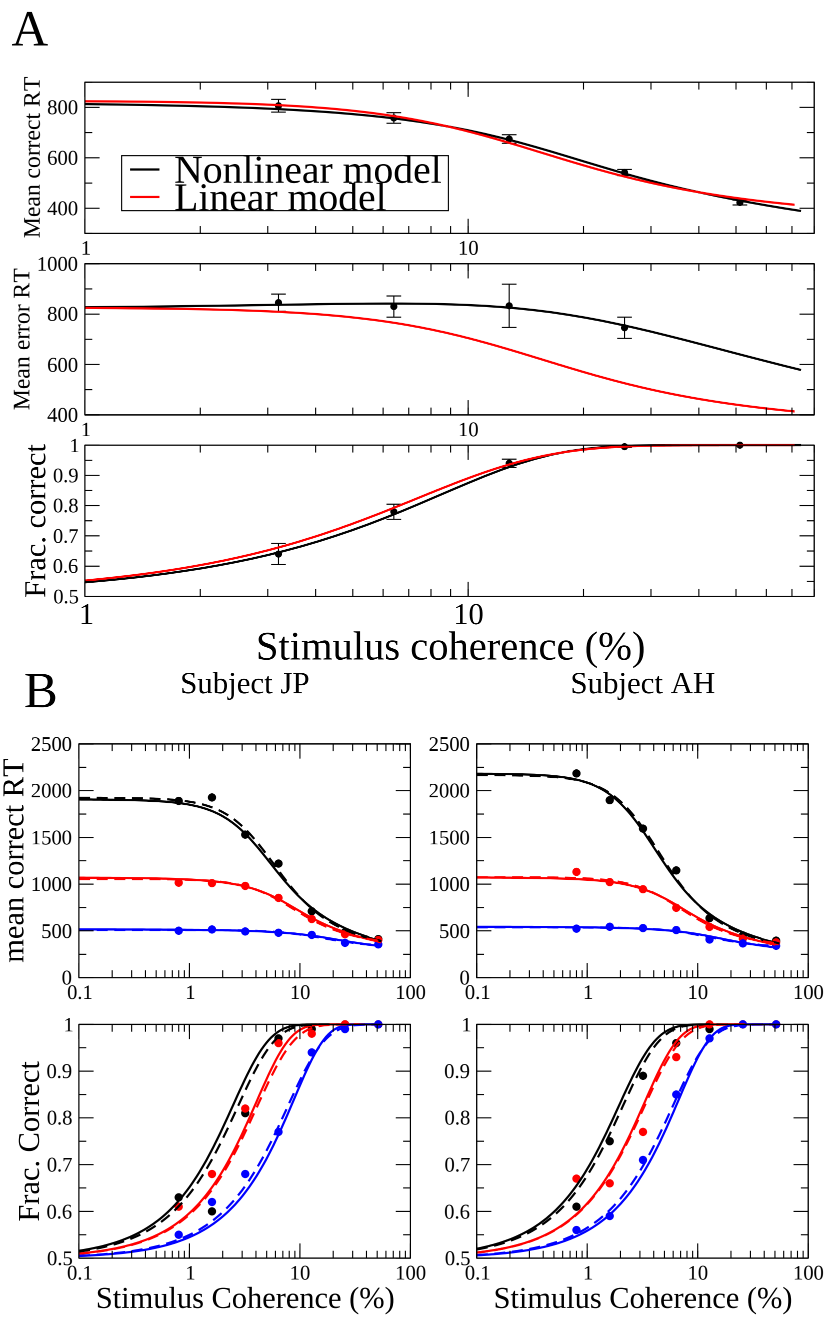

Fig. S.2: A: Fits to data from Roitman and Shadlen 2002, also see Fig.3 in main text. Symbols: experimental data. Black line: fit from nonlinear diffusion equation from main text. Red Line: Fit from linear diffusion equation. B: Fits to data from Palmer et al. 2005, also see Fig.4 in main text. Symbols: experimental data. Solid lines: fit from nonlinear diffusion equation from main text. Dotted lines: Fit from linear diffusion equation.

difference is captured by the nonlinear model. We have not attempted a more quantitative comparison between the models (for example accounting for that the nonlinear model has more parameters) as this is beyond the scope of the present paper. The comparison we have made however indicates that the nonlinear model fits correct RTs and performance data in these two data set about as well as the linear diffusion model does.

2 Derivation of the Amplitude Equation: 2 further examples.

2.1 Example 1: Network of Integrate-and-fire Neurons

We consider a network of recurrently coupled integrate-and-fire neurons. The network architecture consists of two distinct populations of excitatory neurons (A and B), both of which are coupled to a population of inhibitory interneurons. We will study the case in which the system is near a steady bifurcation to a winner-take-all state. In the vicinity of the bifurcation the dynamics can be captured in a one-dimensional amplitude equation which describes the slow evolution along the critical manifold. We follow here the general methodology presented in [2] for a one component system (single population) near a Hopf-bifurcation. We extend that here for a steady bifurcation in a three component system.

The evolution equations for the neuronal membrane potentials are

$$\begin{aligned}
\tau\dot{V}_{A,i} &= -(V_{A,i}-E_e)+I_{AA,i}-I_{AI,i}+I_{Aext,i}, && \text{(S.8)}\\
\tau\dot{V}_{B,i} &= -(V_{B,i}-E_e)+I_{BB,i}-I_{BI,i}+I_{Bext,i}, && \text{(S.9)}\\
\hat{\tau}\dot{V}_{I,i} &= -(V_{I,i}-E_i)+I_{IA,i}+I_{IB,i}+I_{Iext,i}, && \text{(S.10)}
\end{aligned}$$

where the synaptic currents of the form I_{XY} indicate interactions from neurons in population Y to neurons in population X, while external synaptic inputs are given by I_{Xext}. The synaptic currents are sums over all post-synaptic currents (PSCs), modeled as Dirac-delta functions with a delay. The currents take the form

$$\begin{aligned}
I_{XX,i} &= \tau\sum_j J_{ij}^{XX}\sum_k \delta(t-t_{X,j}^k-\delta_{E,ij}), && \text{(S.11)}\\
I_{XI,i} &= \tau\sum_j J_{ij}^{XI}\sum_k \delta(t-t_{I,j}^k-\delta_{I,ij}), && \text{(S.12)}\\
I_{IX,i} &= \hat{\tau}\sum_j J_{ij}^{IX}\sum_k \delta(t-t_{X,j}^k-\delta_{E,ij}), && \text{(S.13)}\\
I_{Xext,i} &= \tau\sum_j J_{ij}^{Xext}\sum_k \delta(t-t_{Xext,j}^k). && \text{(S.14)}
\end{aligned}$$

Given a presynaptic action potential from neuron j of population Y at a time $t_{Y,j}$, there is a resulting jump of size J_{ij}^{XY} in the post-synaptic potential of neuron i from population X after a delay $\delta_{Y,ij}$. A spike is emitted whenever the voltage of a cell from an excitatory (inhibitory) population cross a value θ ($\hat{\theta}$), after which it is reset to a value V_r ($\hat{V}_r$).

We consider the case of sparse connectivity for which, on average, each neuron from population X receives a total of C_{XY} synapses from population Y. The pairwise probability of connection is thus $\epsilon_{XY} = C_{XY}/N_Y$, where $N_A = N_B = N_E$ and N_I are the number of neurons in the respective populations. For nonzero synapses we choose $J_{ij}^{AA} = J_{ij}^{BB} = J_{ee}$, $J_{ij}^{IA} = J_{ij}^{IB} = J_{ie}$, and $J_{ij}^{AI} = J_{ij}^{BI} = J_{ei}$. The resulting network topology exhibits a Poisson degree distribution, i.e. the quenched randomness in the number of connections per cell is Poisson distributed.

If the network activity is asynchronous, then for sufficiently many PSCs the sums over the delta functions can be well approximated by a Poisson process in time with a mean equal to the firing rate of the pre-synaptic population. In this regime the currents due to recurrent excitatory connections can be approximated as

$$I_{XX,i} = \tau J_{ee}\Big(\frac{C_{XX}}{N_E} + \delta C_{i,XX}\Big)\Big(N_E \nu_X(t-\delta_e) + \delta\nu_X(t-\delta_e)\Big), \qquad \text{(S.15)}$$

where δC_{XX} are the quenched fluctuations in the connectivity and $\delta\nu_X$ are the fluctuations in the firing rate of the population. Note that we assume a fixed delay for each neuronal population. Distributions in the delay can be handled through averaging. Retaining only the first order terms in the fluctuations yields

$$I_{XX,i} = \tau J_{ee} C_{XX} \nu_X(t-\delta_e) + \tau J_{ee} \nu_X(t-\delta_e)\delta C_{i,XX} + \tau \epsilon_{XX} J_{ee} \delta\nu_X(t-\delta_e) \qquad \text{(S.16)}$$

The first term in Eq.S.16 is the mean current. The second term represents fluctuations in the current due to the quenched randomness in the connectivity and accounts for differences in input across neurons. Fluctuations at the network level are taken into account by the third term, which is proportional to the small parameter $\epsilon_{XX} = C_{XX}/N_E$. These fluctuations are felt coherently by all neurons in population X. Finally, since the variance of a Poisson process is equal to its mean, we have, for the strength of the fluctuations across neurons $\sigma_{XX}^2 = \tau J_{ee}^2 C_{XX} \nu_X(t-\delta_e)$, whereas the coherent fluctuations have a strength $S_X^2 = \tau \epsilon_{XX} J_{ee}^2 C_{XX} \nu_X(t-\delta_e)$.

If the strength of the synaptic inputs is sufficiently small compared to the firing threshold, then the fluctuations in the input can be approximated as Gaussian distributed. This allows us to write down the Fokker-Planck equation describing the time-evolution of the probability distribution of the membrane voltage across

the network for each population. The equations are

$$\tau \dot{P}_A = \frac{\sigma_A^2(t)}{2}\partial_{V_A}^2 P_A + \partial_{V_A}\left(\left[V_A - \mu_A(t) - S_A\xi_A(t)\right]P_A\right), \tag{S.17}$$

$$\tau \dot{P}_B = \frac{\sigma_B^2(t)}{2}\partial_{V_B}^2 P_B + \partial_{V_B}\left(\left[V_B - \mu_B(t) - S_B\xi_B(t)\right]P_B\right), \tag{S.18}$$

$$\hat{\tau} \dot{P}_I = \frac{\sigma_I^2(t)}{2}\partial_{V_I}^2 P_I + \partial_{V_I}\left(\left[V_I - \mu_I(t) - S_I\xi_I(t)\right]P_I\right), \tag{S.19}$$

where the mean inputs are

$$\mu_A = \tau J_{ee}C_{ee}\nu_A(t-\delta_e) - \tau J_{ei}C_{ei}\nu_I(t-\delta_i) + \tau J_{ext}C_{ext}\nu_{Aext} + E_e, \tag{S.20}$$

$$\mu_B = \tau J_{ee}C_{ee}\nu_B(t-\delta_e) - \tau J_{ei}C_{ei}\nu_I(t-\delta_i) + \tau J_{ext}C_{ext}\nu_{Bext} + E_e, \tag{S.21}$$

$$\mu_I = \tau J_{ie}C_{ie}\Big(\nu_A(t-\delta_e) + \nu_B(t-\delta_e)\Big) + \tau J_{ext}C_{ext}\nu_{Iext} + E_i, \tag{S.22}$$

the variances in the inputs are

$$\sigma_A^2 = \tau J_{ee}^2 C_{ee}\nu_A(t-\delta_e) + \tau J_{ei}^2 C_{ei}\nu_I(t-\delta_i) + \tau J_{ext}^2 C_{ext}\nu_{Aext}, \tag{S.23}$$

$$\sigma_B^2 = \tau J_{ee}^2 C_{ee}\nu_B(t-\delta_e) + \tau J_{ei}^2 C_{ei}\nu_I(t-\delta_i) + \tau J_{ext}^2 C_{ext}\nu_{Bext}, \tag{S.24}$$

$$\sigma_I^2 = \tau J_{ie}^2 C_{ie}\Big(\nu_A(t-\delta_e) + \nu_B(t-\delta_e)\Big) + \tau J_{ext}^2 C_{ext}\nu_{Iext}, \tag{S.25}$$

and the strengths of the coherent fluctuations are

$$S_A^2 = \tau\epsilon_{ee}J_{ee}^2 C_{ee}\nu_A(t-\delta_e) + \tau\epsilon_{ei}J_{ei}^2 C_{ei}\nu_I(t-\delta_i), \tag{S.26}$$

$$S_B^2 = \tau\epsilon_{ee}J_{ee}^2 C_{ee}\nu_B(t-\delta_e) + \tau\epsilon_{ei}J_{ei}^2 C_{ei}\nu_I(t-\delta_i), \tag{S.27}$$

$$S_I^2 = \tau\epsilon_{ie}J_{ie}^2 C_{ee}\Big(\nu_A(t-\delta_e) + \nu_B(t-\delta_e)\Big). \tag{S.28}$$

Note that for simplicity we have taken $C_{AA} = C_{BB} = C_{ee}$, $C_{AI} = C_{BI} = C_{ei}$ and $C_{IA} = C_{IB} = C_{ie}$. Also we note that $S_A = S_B = S_E$

The boundary conditions for population A are

$$P_A(\theta, t) = \Big[P_A(V_A, t)\Big]_{V_r^-}^{V_r^+} = 0, \tag{S.29}$$

$$P_A'(\theta, t) = \Big[P_A'(V_A, t)\Big]_{V_r^-}^{V_r^+} = -\frac{2\tau\nu_A(t)}{\sigma_A^2}, \tag{S.30}$$

$$\int_{-\infty}^{\theta} du P_A(u, t) = 1, \tag{S.31}$$

with analogous conditions for the other two populations.

2.1.1 Stationary Distributions

The steady-state distributions of the membrane voltages can be found by setting the left hand side equal to zero in Eqs(S.17) through (S.19) and solving together with the boundary conditions (BCs) Eqs(S.29) through (S.31), in which the time dependence is removed. The stationary voltage-distribution of population A is

$$P_A(V) = \frac{2\bar{\nu}_A\tau}{\bar{\sigma}_A} e^{-\frac{(V-\bar{\mu}_A)^2}{\bar{\sigma}_A^2}} \int_{\frac{V-\bar{\mu}_A}{\bar{\sigma}_A}}^{\frac{\theta-\bar{\mu}_A}{\bar{\sigma}_A}} du e^{u^2} H\left(u - \frac{V_r - \bar{\mu}_A}{\bar{\sigma}_A}\right) \tag{S.32}$$

where $\bar{\nu}_A$ is the steady-state firing rate of population A, and the steady-state values of the mean input and variance can be found by plugging the steady-state firing rates into Eqs(S.20) through (S.25). Analogous expressions hold for populations B and I. $H(x)$ is the Heaviside function. The mean firing rates of the populations are given by the following relations

$$\sqrt{\pi}\bar{\nu}_X\tau \int_{\frac{V_r-\bar{\mu}_X}{\bar{\sigma}_X}}^{\frac{\theta-\bar{\mu}_X}{\bar{\sigma}_X}} du e^{u^2} \mathrm{erfc}(-u) = 1, \tag{S.33}$$

where $X = A, B$, and I [2]. The coefficient of variation (CV) of the inter-spike interval can also be shown to be

$$\mathrm{CV} = 2\pi\bar{\nu}_X^2\tau^2 \int_{\frac{V_r-\bar{\mu}_X}{\bar{\sigma}_X}}^{\frac{\theta-\bar{\mu}_X}{\bar{\sigma}_X}} dv e^{v^2} \int_{-\infty}^{v} du e^{u^2} [\mathrm{erfc}(-u)]^2, \tag{S.34}$$

[1].

2.1.2 Linear Stability and Weakly Nonlinear Analysis

Since we are interested in investigating the network dynamics for a discrimination task, i.e. $\nu_{Aext} \sim \nu_{Bext}$, we assume that differences in the afferent firing rates to populations A and B are small. We can then take the mean firing rates to be $\bar{\nu}_A = \bar{\nu}_B = \bar{\nu}_E$ to leading order.

For convenience we write $P_A = \frac{2\tau\bar{\nu}_A}{\bar{\sigma}_A} Q_A$, $y_A = \frac{V_A-\bar{\mu}_A}{\bar{\sigma}_A}$ and $\nu_A = \bar{\nu}_E(1 + n_A(t))$ and analogously for populations B and I. Eqs (S.17) through (S.19) can then be

rewritten as

$$
\begin{aligned}
\tau \dot{Q}_A &= \mathcal{L}Q_A + n_A(t-\delta_e)\Bigg(- G_{ee}Q_A^{'} + \frac{H_{ee}}{2}Q_A^{''}\Bigg) \\
&\quad + n_I(t-\delta_i)\Bigg(G_{ei}Q_A^{'} + \frac{H_{ei}}{2}Q_A^{''}\Bigg) - \frac{S_A}{\bar{\sigma_A}}\xi_A(t)Q_A^{'}, \qquad \text{(S.35)}\\
\tau \dot{Q}_B &= \mathcal{L}Q_B + n_B(t-\delta_e)\Bigg(- G_{ee}Q_B^{'} + \frac{H_{ee}}{2}Q_B^{''}\Bigg) \\
&\quad + n_I(t-\delta_i)\Bigg(G_{ei}Q_B^{'} + \frac{H_{ei}}{2}Q_B^{''}\Bigg) - \frac{S_B}{\bar{\sigma_B}}\xi_B(t)Q_B^{'}, \qquad \text{(S.36)}\\
\hat{\tau} \dot{Q}_I &= \mathcal{L}Q_I + \Big(n_A(t-\delta_e) + n_B(t-\delta_e)\Big)\Bigg(- G_{ie}Q_A^{'} + \frac{H_{ie}}{2}Q_A^{''}\Bigg) \\
&\quad - \frac{S_I}{\bar{\sigma_I}}\xi_I(t)Q_I^{'}, \qquad \text{(S.37)}
\end{aligned}
$$

where we have written $G_{ee} = \frac{\tau J_{ee}C_{ee}\bar{\nu_e}}{\bar{\sigma}_e}$, $G_{ei} = \frac{\tau J_{ei}C_{ei}\bar{\nu_I}}{\bar{\sigma}_e}$, $G_{ie} = \frac{\tau J_{ie}C_{ie}\bar{\nu_e}}{\bar{\sigma}_I}$, $H_{ee} = \frac{\tau J_{ee}^2 C_{ee}\bar{\nu_e}}{\bar{\sigma}_e^2}$, $H_{ei} = \frac{\tau J_{ei}^2 C_{ei}\bar{\nu_I}}{\bar{\sigma}_e^2}$, $H_{ie} = \frac{\tau J_{ie}^2 C_{ie}\bar{\nu_e}}{\bar{\sigma}_I^2}$. The BCs for population A are

$$
\begin{aligned}
Q_A(y_\theta) = \Big[Q_A(y_A,t)\Big]_{y_r^-}^{y_r^+} &= 0 \qquad \text{(S.38)}\\
Q_A^{'}(y_\theta) = \Big[Q_A^{'}(y_A,t)\Big]_{y_r^-}^{y_r^+} &= -\frac{(1+n_A(t))}{1+H_{ee}n_A(t-\delta_e)+H_{ei}n_I(t-\delta_i)} \qquad \text{(S.39)}\\
\int_{-\infty}^{y_\theta} du Q_A(u,t) &= \frac{1}{2\tau\bar{\nu}_A} \qquad \text{(S.40)}
\end{aligned}
$$

and analogous conditions hold for populations B and I.

We are now interested in determining the critical value of the external, afferent inputs to populations A and B for which a steady bifurcation occurs leading to a winner-take-all scenario. We then wish to extend the linear stability calculation to include nonlinear effects, thereby deriving an evolution equation for the winner-take-all dynamics near the bifurcation.

We assume that the bifurcation occurs for an input $\nu_{Aext} = \nu_{Bext} = \bar{\nu}_{Eext}$ and $\nu_{Iext} = \bar{\nu}_{Iext}$. We then express the inputs as

$$
\begin{aligned}
\nu_{Aext} &= \bar{\nu}_{Eext}\Big(1+\epsilon^2\Delta\nu_E + \epsilon^3\Delta\nu_A\Big), \qquad \text{(S.41)}\\
\nu_{Bext} &= \bar{\nu}_{Eext}\Big(1+\epsilon^2\Delta\nu_E + \epsilon^3\Delta\nu_B\Big), \qquad \text{(S.42)}\\
\nu_{Iext} &= \bar{\nu}_{Iext}\Big(1+\epsilon^2\Delta\nu_I\Big), \qquad \text{(S.43)}
\end{aligned}
$$

where ϵ is a small parameter which measures the distance from the bifurcation. Thus the difference in the afferent inputs to both populations is proportional to $\nu_A - \nu_B$. We now expand the voltage distributions and mean firing rates in ϵ

$$Q_A(y_A, t) = Q_E(y_A) + \epsilon Q_{A1}(y_A, T) + \epsilon^2 Q_{A2}(y_A, T) + \ldots, \quad \text{(S.44)}$$
$$n_A(t) = \epsilon n_{A1}(T) + \epsilon^2 n_{A2}(T) + \ldots, \quad \text{(S.45)}$$

and similar expansions hold for populations B and I. Here we have introduced a slow time T, where $T = \epsilon^2 t$.

We can now plug the expansions, Eqs.(S.44) through (S.45), into Eqs.(S.35) and the boundary conditions Eqs.(S.38) through (S.40), and similarly for populations B and I. Grouping terms by their order in ϵ yields a systems of equations at each order which we must solve.

2.1.3 Stationary distributions $\vartheta(1)$

To leading order we regain the stationary distributions given by the system

$$\mathcal{L}Q_E = 0, \quad \text{(S.46)}$$
$$\mathcal{L}Q_I = 0, \quad \text{(S.47)}$$

with boundary conditions

$$Q_E(y_\theta) = \Big[Q_E\Big]_{y_r^-}^{y_r^+} = 0, \quad \text{(S.48)}$$
$$Q_E^{'}(y_\theta) = \Big[Q_E^{'}\Big]_{y_r^-}^{y_r^+} = -1, \quad \text{(S.49)}$$
$$\int_{-\infty}^{y_\theta} du Q_E(u) = \frac{1}{2\tau\bar{\nu_E}}, \quad \text{(S.50)}$$
$$Q_I(\hat{y}_\theta) = \Big[Q_I\Big]_{\hat{y}_r^-}^{\hat{y}_r^+} = 0, \quad \text{(S.51)}$$
$$Q_I^{'}(\hat{y}_\theta) = \Big[Q_I^{'}\Big]_{\hat{y}_r^-}^{\hat{y}_r^+} = -1, \quad \text{(S.52)}$$
$$\int_{-\infty}^{\hat{y}_\theta} du Q_I(u) = \frac{1}{2\tau\bar{\nu_I}}, \quad \text{(S.53)}$$

where $\mathcal{L} = \frac{1}{2}\partial_y^2 + y\partial_y + 1$.

The solutions are

$$Q_E(y) = \begin{cases} e^{-y^2} \int_y^{y_\theta} due^{u^2} & y > y_r \\ e^{-y^2} \int_{y_r}^{y_\theta} due^{u^2} & y < y_r \end{cases} \quad \text{(S.54)}$$

$$Q_I(y) = \begin{cases} e^{-y^2} \int_y^{\hat{y}_\theta} due^{u^2} & y > \hat{y}_r, \\ e^{-y^2} \int_{\hat{y}_r}^{\hat{y}_\theta} due^{u^2} & y < \hat{y}_r. \end{cases} \quad \text{(S.55)}$$

2.1.4 Linear Stability $\vartheta(\epsilon)$

The equations are

$$\begin{aligned}
\mathcal{L}Q_{A1} &= -n_{A1}\Bigg(- G_{ee}Q_E^{'} + \frac{H_{ee}}{2}Q_E^{''}\Bigg) - n_{I1}\Bigg(G_{ei}Q_E^{'} + \frac{H_{ei}}{2}Q_E^{''}\Bigg), \quad \text{(S.56)}\\
\mathcal{L}Q_{B1} &= -n_{B1}\Bigg(- G_{ee}Q_E^{'} + \frac{H_{ee}}{2}Q_E^{''}\Bigg) - n_{I1}\Bigg(G_{ei}Q_E^{'} + \frac{H_{ei}}{2}Q_E^{''}\Bigg), \quad \text{(S.57)}\\
\mathcal{L}Q_{I1} &= -(n_{A1} + n_{B1})\Bigg(- G_{ie}Q_I^{'} + \frac{H_{ie}}{2}Q_I^{''}\Bigg), \quad \text{(S.58)}
\end{aligned}$$

with boundary conditions

$$\begin{aligned}
Q_{A1}(y_\theta) = \Big[Q_{A1}\Big]_{y_r^-}^{y_r^+} &= 0, \quad \text{(S.59)}\\
Q_{A1}^{'}(y_\theta) = \Big[Q_{A1}^{'}\Big]_{y_r^-}^{y_r^+} &= (H_{ee} - 1)n_{A1} + H_{ei}n_{I1}, \quad \text{(S.60)}\\
\int_{-\infty}^{y_\theta} duQ_{A1}(u) &= 0, \quad \text{(S.61)}\\
Q_{B1}(y_\theta) = \Big[Q_{B1}\Big]_{y_r^-}^{y_r^+} &= 0, \quad \text{(S.62)}\\
Q_{B1}^{'}(y_\theta) = \Big[Q_{B1}^{'}\Big]_{y_r^-}^{y_r^+} &= (H_{ee} - 1)n_{B1} + H_{ei}n_{I1}, \quad \text{(S.63)}\\
\int_{-\infty}^{y_\theta} duQ_{B1}(u) &= 0, \quad \text{(S.64)}\\
Q_{I1}(\hat{y}_\theta) = \Big[Q_{I1}\Big]_{\hat{y}_r^-}^{\hat{y}_r^+} &= 0, \quad \text{(S.65)}\\
Q_{I1}^{'}(\hat{y}_\theta) = \Big[Q_{I1}^{'}\Big]_{\hat{y}_r^-}^{\hat{y}_r^+} &= H_{ie}(n_{A1} + n_{B1}) - n_{I1}, \quad \text{(S.66)}\\
\int_{-\infty}^{\hat{y}_\theta} duQ_{I1}(u) &= 0. \quad \text{(S.67)}
\end{aligned}$$

Eqs.(S.56) through (S.58) are inhomogeneous second-order equations. Their solution therefore consists of two independent solutions of the homogeneous equation plus a particular solution. We can choose the stationary distribution as one of the two homogeneous solutions, whereas it is convenient to choose the other as

e^{-y^2}. Then, utilizing the boundary conditions Eqs.(S.59) through (S.67) yields

$$Q_{A1}(y) = \begin{cases} n_{A1}Q_E(y) - e^{y_\theta^2}Q^p_{A1}(y_\theta)e^{-y^2} + Q^p_{A1}(y) & y > y_r, \\ n_{A1}Q_E(y) + \Big(- e^{y_\theta^2}Q^p_{A1}(y_\theta) + e^{y_r^2}[Q^p_{A1}]^{y_r^+}_{y_r^-}\Big)e^{-y^2} + Q^p_{A1}(y) & y < y_r, \end{cases} \quad \text{(S.68)}$$

$$Q_{B1}(y) = \begin{cases} n_{B1}Q_E(y) - e^{y_\theta^2}Q^p_{B1}(y_\theta)e^{-y^2} + Q^p_{B1}(y) & y > y_r, \\ n_{B1}Q_E(y) + \Big(- e^{y_\theta^2}Q^p_{B1}(y_\theta) + e^{y_r^2}[Q^p_{B1}]^{y_r^+}_{y_r^-}\Big)e^{-y^2} + Q^p_{B1}(y) & y < y_r, \end{cases} \quad \text{(S.69)}$$

$$Q_{I1}(y) = \begin{cases} n_{I1}Q_I(y) - e^{\hat{y}_\theta^2}Q^p_{I1}(\hat{y}_\theta)e^{-y^2} + Q^p_{I1}(y) & y > \hat{y}_r, \\ n_{I1}Q_I(y) + \Big(- e^{\hat{y}_\theta^2}Q^p_{I1}(\hat{y}_\theta) + e^{\hat{y}_r^2}[Q^p_{I1}]^{\hat{y}_r^+}_{\hat{y}_r^-}\Big)e^{-y^2} + Q^p_{I1}(y) & y < \hat{y}_r, \end{cases} \quad \text{(S.70)}$$

where the particular solutions are

$$Q^p_{A1} = n_{A1}\Bigg(- G_{ee}Q_E^{'} + \frac{1}{4}H_{ee}Q_E^{''}\Bigg) + n_{I1}\Bigg(G_{ei}Q_E^{'} + \frac{1}{4}H_{ei}Q_E^{''}\Bigg), \quad \text{(S.71)}$$

$$Q^p_{B1} = n_{B1}\Bigg(- G_{ee}Q_E^{'} + \frac{1}{4}H_{ee}Q_E^{''}\Bigg) + n_{I1}\Bigg(G_{ei}Q_E^{'} + \frac{1}{4}H_{ei}Q_E^{''}\Bigg), \quad \text{(S.72)}$$

$$Q^p_{I1} = (n_{A1} + n_{B1})\Bigg(- G_{ie}Q_I^{'} + \frac{1}{4}H_{ie}Q_I^{''}\Bigg). \quad \text{(S.73)}$$

The normalization conditions Eqs.(S.61), (S.64) and (S.67) lead to the following matrix equation

$$\left(\begin{array}{ccc} \frac{1}{2\tau\bar{\nu}_E} - \Gamma(G_{ee},H_{ee}) & 0 & \Gamma(G_{ei},-H_{ei}) \\ 0 & \frac{1}{2\tau\bar{\nu}_E} - \Gamma(G_{ee},H_{ee}) & \Gamma(G_{ei},-H_{ei}) \\ -\Gamma(G_{ie},H_{ie}) & -\Gamma(G_{ie},H_{ie}) & \frac{1}{2\hat{\tau}\bar{\nu}_I} \end{array}\right)\left(\begin{array}{c} n_{A1} \\ n_{B1} \\ n_{I1}\end{array}\right) = 0, \quad \text{(S.74)}$$

where

$$\Gamma(f_1,f_2) = \frac{\sqrt{\pi}}{2}\Bigg[\Big(f_1 + \frac{y}{2}f_2\Big)e^{y^2}\mathrm{erfc}(-y)\Bigg]^{y_\theta}_{y_r}. \quad \text{(S.75)}$$

A solution to Eq.(S.74) will only exist if the determinant of the matrix is equal to zero, which requires that $\Gamma(G_{ee},H_{ee}) = \frac{1}{2\tau\bar{\nu}_E}$. This relationship then sets the critical value of the control parameter, e.g. ν_{Eext}, for which a steady bifurcation occurs.

The solution is then given by $(n_{A1}, n_{B1}, n_{I1}) = (1,-1,0)X(T) = \mathbf{n_c}X(T)$, where $X(T)$ is an unknown amplitude which is assumed to vary slowly in time.

We note at this point that any vector in the phase space of the three component system can be expressed as a linear combination of the mutually orthogonal vectors $\mathbf{n_c} = (1,-1,0)$, $\mathbf{n_{s1}} = (1,1,1)$ and $\mathbf{n_{s2}} = (1,1,-2)$. We also note that the left-null eigenvector of Eq.(S.74) can be written $\mathbf{n}^\dagger = (1,-1,0)$ and that the left eigenspace is thus spanned by the same above-mentioned vectors.

2.1.5 $\vartheta(\epsilon^2)$

The equations are

$$\begin{aligned}
\mathcal{L}Q_{A2} &= -n_{A2}\Bigg(-G_{ee}Q_E^{'}+\frac{H_{ee}}{2}Q_E^{''}\Bigg)-n_{I2}\Bigg(G_{ei}Q_E^{'}+\frac{H_{ei}}{2}Q_E^{''}\Bigg)\\
&\quad -X(T)\Bigg(-G_{ee}Q_{A1}^{'}+\frac{H_{ee}}{2}Q_{A1}^{''}\Bigg)-\Delta\nu_E\Bigg(-B_eQ_E^{'}+\frac{D_e}{2}Q_E^{''}\Bigg), \quad \text{(S.76)}\\
\mathcal{L}Q_{B2} &= -n_{B2}\Bigg(-G_{ee}Q_E^{'}+\frac{H_{ee}}{2}Q_E^{''}\Bigg)-n_{I2}\Bigg(G_{ei}Q_E^{'}+\frac{H_{ei}}{2}Q_E^{''}\Bigg)\\
&\quad X(T)\Bigg(-G_{ee}Q_{B1}^{'}+\frac{H_{ee}}{2}Q_{B1}^{''}\Bigg)-\Delta\nu_E\Bigg(-B_eQ_E^{'}+\frac{D_e}{2}Q_E^{''}\Bigg), \quad \text{(S.77)}\\
\mathcal{L}Q_{I2} &= -(n_{A2}+n_{B2})\Bigg(-G_{ie}Q_I^{'}+\frac{H_{ie}}{2}Q_I^{''}\Bigg)-\Delta\nu_I\Bigg(-B_iQ_I^{'}+\frac{D_i}{2}Q_I^{''}\Bigg) \quad \text{(S.78)}
\end{aligned}$$

with boundary conditions

$$\begin{aligned}
Q_{A2}(y_\theta)=\Big[Q_{A2}\Big]_{y_r^-}^{y_r^+} &= 0, && \text{(S.79)}\\
Q_{A2}^{'}(y_\theta)=\Big[Q_{A2}^{'}\Big]_{y_r^-}^{y_r^+} &= (H_{ee}-1)n_{A2}+H_{ei}n_{I2}+X^2(H_{ee}-H_{ee}^2)\\
&\quad +D_e\Delta\nu_E, && \text{(S.80)}\\
\int_{-\infty}^{y_\theta} duQ_{A2}(u) &= 0, && \text{(S.81)}\\
Q_{B2}(y_\theta)=\Big[Q_{B2}\Big]_{y_r^-}^{y_r^+} &= 0, && \text{(S.82)}\\
Q_{B2}^{'}(y_\theta)=\Big[Q_{B2}^{'}\Big]_{y_r^-}^{y_r^+} &= (H_{ee}-1)n_{B2}+H_{ei}n_{I2}+X^2(H_{ee}-H_{ee}^2)\\
&\quad +D_e\Delta\nu_E, && \text{(S.83)}\\
\int_{-\infty}^{y_\theta} duQ_{B2}(u) &= 0, && \text{(S.84)}\\
Q_{I2}(\bar{y}_\theta)=\Big[Q_{I2}\Big]_{\bar{y}_r^-}^{\bar{y}_r^+} &= 0, && \text{(S.85)}\\
Q_{I2}^{'}(\bar{y}_\theta)=\Big[Q_{I2}^{'}\Big]_{\bar{y}_r^-}^{\bar{y}_r^+} &= H_{ie}(n_{A2}+n_{B2})-n_{I2}+D_i\Delta\nu_I, && \text{(S.86)}\\
\int_{-\infty}^{\bar{y}_\theta} duQ_{I2}(u) &= 0, && \text{(S.87)}
\end{aligned}$$

where $B_e=\frac{\tau J_{Eext}C_{Eext}\bar{\nu}_{Eext}}{\bar{\sigma}_E}$, $D_e=\frac{B_eJ_{Eext}}{\bar{\sigma}_E}$, $B_i=\frac{\bar{\tau}J_{Iext}C_{Iext}\bar{\nu}_{Iext}}{\bar{\sigma}_I}$, and $D_i=\frac{B_iJ_{Iext}}{\bar{\sigma}_I}$.

The voltage distributions at this order are given by

$$Q_{A2}(y) = \begin{cases} \alpha_{A2}Q_E(y) + \beta^+_{A2}e^{-y^2} + Q^{p0}_{A2}(y) + Q^{p1}_{A2}(y) & y > y_r \\ \alpha_{A2}Q_E(y) + \beta^-_{A2}e^{-y^2} + Q^{p0}_{A2}(y) + Q^{p1}_{A2}(y) & y < y_r, \end{cases} \quad \text{(S.88)}$$

$$Q_{I2}(y) = \begin{cases} \alpha_{I2}Q_I(y) + \beta^+_{I2}e^{-y^2} + Q^{p0}_{I2}(y) + Q^{p1}_{I2}(y) & y > \hat{y}_r \\ \alpha_{I2}Q_I(y) + \beta^-_{I2}e^{-y^2} + Q^{p0}_{I2}(y) + Q^{p1}_{I2}(y) & y < \hat{y}_r, \end{cases} \quad \text{(S.89)}$$

where

$$Q^{p0}_{A2} = n_{A2}\left(- G_{ee}Q^{'}_E + \frac{1}{4}H_{ee}Q^{''}_E\right) + n_{I2}\left(G_{ei}Q^{'}_E + \frac{1}{4}H_{ei}Q^{''}_E\right), \quad \text{(S.90)}$$

$$Q^{p1}_{A2} = -XG_{ee}Q^{'}_{A1} + \frac{X}{4}H_{ee}Q^{''}_{A1} - B_e\Delta\nu_E Q^{'}_E + \left(- \frac{X^2}{2}G^2_{ee} + \frac{1}{4}D_e\Delta\nu_E\right)Q^{''}_E + \frac{X^2}{4}G_{ee}H_{ee}Q^{'''}_E - \frac{X^2}{32}H^2_{ee}Q^{(iv)}_E, \quad \text{(S.91)}$$

$$\alpha_{A2} = n_{A2} \quad \text{(S.92)}$$

$$\beta^+_{A2} = -e^{y^2_\theta}Q^{p0}_{A2}(y_\theta) - e^{y^2_\theta}Q^{p1}_{A2}(y_\theta), \quad \text{(S.93)}$$

$$\beta^-_{A2} = \beta^+_{A2} + e^{y^2_r}[Q^{p0}_{A2}]^{y^+_r}_{y^-_r} + e^{y^2_r}[Q^{p1}_{A2}]^{y^+_r}_{y^-_r}, \quad \text{(S.94)}$$

$$Q^{p0}_{I2} = (n_{A2} + n_{B2})\left(- G_{ie}Q^{'}_I + \frac{1}{4}H_{ie}Q^{''}_I\right), \quad \text{(S.95)}$$

$$Q^{p1}_{I2} = \left(- B_iQ^{'}_I + \frac{1}{4}D_iQ^{''}_I\right)\Delta\nu_I, \quad \text{(S.96)}$$

$$\alpha_{I2} = n_{I2}, \quad \text{(S.97)}$$

$$\beta^+_{I2} = -e^{\bar{y}^2_\theta}Q^{p0}_{I2}(\bar{y}_\theta) - e^{\bar{y}^2_\theta}Q^{p1}_{I2}(\bar{y}_\theta), \quad \text{(S.98)}$$

$$\beta^-_{I2} = \beta^+_{I2} + e^{\bar{y}^2_r}[Q^{p0}_{I2}]^{\bar{y}^+_r}_{\bar{y}^-_r} + e^{\bar{y}^2_r}[Q^{p1}_{I2}]^{\bar{y}^+_r}_{\bar{y}^-_r} \quad \text{(S.99)}$$

and it can be shown that $Q_{B2}(y) = Q_{A2}(y)$ at this order.

The normalization conditions, Eqs.(S.81), (S.84) and (S.87) lead to the matrix equation

$$\mathbb{L}\mathbf{n_2} = \mathbf{N_2} \quad \text{(S.100)}$$

where

$$\mathbb{L} = \begin{pmatrix} 0 & 0 & \Gamma_{ei} \\ 0 & 0 & \Gamma_{ei} \\ \Gamma_{ie} & \Gamma_{ie} & \frac{1}{2\hat{\tau}\bar{\nu}_I} \end{pmatrix}, \tag{S.101}$$

$$\mathbf{N_2} = \begin{pmatrix} AX^2 + \Gamma(B_e, D_e)\Delta\nu_E \\ AX^2 + \Gamma(B_e, D_e)\Delta\nu_E \\ \Gamma(B_i, D_i)\Delta\nu_I \end{pmatrix}, \tag{S.102}$$

and

$$\begin{aligned} A &= \Gamma(G_{ee}, H_{ee}) - \frac{\sqrt{\pi}}{2}\left[\left(yG_{ee}^2 + (\frac{1}{2} + y^2)G_{ee}H_{ee} + \frac{1}{8}(3y + 2y^3)H_{ee}^2\right)e^{y^2}\text{erfc}(-y)\right]_{y_r}^{y_\theta} \\ &\quad - \frac{(y_\theta^2 - y_r^2)}{8}H_{ee}^2 - \frac{(y_\theta - y_r)}{2}G_{ee}H_{ee}. \end{aligned} \tag{S.103}$$

The matrix $\mathbb{L}$ can be recognized as that arising in the linear problem at the bifurcation point, see Eq.(S.74), and is thus clearly not invertible. There exists a solution to Eq.(S.100) only if $\mathbf{N_2}$ lies outside of the left-null eigenspace of the matrix, i.e. $\mathbf{n}^\dagger \cdot \mathbf{N_2} = 0$. It is clear that this condition is met upon inspection.

The firing rates at this order can then be expressed as $\mathbf{n_2} = R_1\mathbf{n_{s1}} + R_2\mathbf{n_{s2}}$ which can be equivalently written as $\mathbf{n_2} = (\Pi_1, \Pi_1, \Pi_2)$ where $\Pi_1 = R_1 + R_2$ and $\Pi_2 = R_1 - 2R_2$. The coefficients Π_1 and Pi_2 can be determined by projecting Eq.(S.100) onto the eigenspace orthogonal to the left-null eigenspace of the operator $\mathbb{L}$. This is equivalent to operating on Eq.(S.100) from the left with $\mathbf{n_{s1}}$ and $\mathbf{n_{s2}}$. Doing so leads to the system of equations

$$\begin{pmatrix} -2\Gamma(G_{ie}, H_{ie}) & 2\Gamma(G_{ei}, -H_{ei}) + \frac{1}{2\hat{\tau}\bar{\nu}_I} \\ 4\Gamma(G_{ie}, H_{ie}) & 2\Gamma(G_{ei}, -H_{ei}) - \frac{1}{\hat{\tau}\bar{\nu}_I} \end{pmatrix}\begin{pmatrix} \Pi_1 \\ \Pi_2 \end{pmatrix} = \begin{pmatrix} 2AX^2 + 2\Gamma(B_e, D_e)\Delta\nu_E + \Gamma(B_i, D_i)\Delta\nu_I \\ 2AX^2 + 2\Gamma(B_e, D_e)\Delta\nu_E - 2\Gamma(B_i, D_i)\Delta\nu_I \end{pmatrix}, \tag{S.104}$$

the solution of which is

$$\begin{aligned} \Pi_1 &= \frac{A}{4\Gamma(G_{ie}, H_{ie})\Gamma(G_{ei}, -H_{ei})\hat{\tau}\nu_I}X^2 + \frac{\Gamma(B_e, D_e)}{4\Gamma(G_{ie}, H_{ie})\Gamma(G_{ei}, -H_{ei})\hat{\tau}\nu_I}\Delta\nu_E - \frac{\Gamma(B_i, D_i)}{2\Gamma(G_{ie}, H_{ie})}\Delta\nu_I \\ \Pi_2 &= \frac{A}{\Gamma(G_{ei}, -H_{ei})}X^2 + \frac{\Gamma(B_e, D_e)}{\Gamma(G_{ei}, -H_{ei})}\Delta\nu_E \end{aligned} \tag{S.105}$$

Furthermore, we can write $\Pi_j = \Pi_{jI}\Delta\nu_I + \Pi_{jE}\Delta\nu_E + \Pi_{jC}X^2$ for $j = 1, 2$.

2.1.6 $\vartheta(\epsilon^3)$

The equations are

$$\begin{aligned}
\mathcal{L}Q_{A3} &= -n_{A3}\left(-G_{ee}Q_E^{'}+\frac{1}{2}H_{ee}Q_E^{''}\right)-n_{I3}\left(-G_{ei}Q_E^{'}+\frac{1}{2}H_{ei}Q_E^{''}\right)\\
&-\Pi_1\left(-G_{ee}Q_{A1}^{'}+\frac{1}{2}H_{ee}Q_{A1}^{''}\right)-\Pi_2\left(G_{ei}Q_{A1}^{'}+\frac{1}{2}H_{ei}Q_{A1}^{''}\right)\\
&-X\left(-G_{ee}Q_{A2}^{'}+\frac{1}{2}H_{ee}Q_{A2}^{''}\right)+\delta_e\partial_T X\left(-G_{ee}Q_E^{'}+\frac{1}{2}H_{ee}Q_E^{''}\right)\\
&-\Delta\nu_E\left(-B_eQ_{A1}^{'}+\frac{1}{2}D_eQ_{A1}^{''}\right)-\Delta\nu_A\left(-B_eQ_E^{'}+\frac{1}{2}D_eQ_E^{''}\right)\\
&+\tau\partial_T Q_{A1}+\frac{S_E}{\bar{\sigma}_E}\xi_A Q_E^{'} \qquad \text{(S.106)}\\
\mathcal{L}Q_{B3} &= -n_{B3}\left(-G_{ee}Q_E^{'}+\frac{1}{2}H_{ee}Q_E^{''}\right)-n_{I3}\left(-G_{ei}Q_E^{'}+\frac{1}{2}H_{ei}Q_E^{''}\right)\\
&-\Pi_1\left(-G_{ee}Q_{B1}^{'}+\frac{1}{2}H_{ee}Q_{B1}^{''}\right)-\Pi_2\left(G_{ei}Q_{B1}^{'}+\frac{1}{2}H_{ei}Q_{B1}^{''}\right)\\
&+X\left(-G_{ee}Q_{B2}^{'}+\frac{1}{2}H_{ee}Q_{B2}^{''}\right)-\delta_e\partial_T X\left(-G_{ee}Q_E^{'}+\frac{1}{2}H_{ee}Q_E^{''}\right)\\
&-\Delta\nu_E\left(-B_eQ_{B1}^{'}+\frac{1}{2}D_eQ_{B1}^{''}\right)-\Delta\nu_B\left(-B_eQ_E^{'}+\frac{1}{2}D_eQ_E^{''}\right)\\
&+\tau\partial_T Q_{B1}+\frac{S_E}{\bar{\sigma}_E}\xi_B Q_E^{'} \qquad \text{(S.107)}\\
\mathcal{L}Q_{I3} &= -(n_{A3}+n_{B3})\left(-G_{ie}Q_I^{'}+\frac{1}{2}H_{ie}Q_I^{''}\right)-2\Pi_1\left(-G_{ie}Q_{I1}^{'}+\frac{1}{2}H_{ie}Q_{I1}^{''}\right)\\
&+\hat{\tau}\partial_T Q_{I1}+\frac{S_I}{\bar{\sigma}_I}\xi_I Q_I^{'} \qquad \text{(S.108)}
\end{aligned}$$

with boundary conditions

$$\begin{aligned}
Q_{A3}(y_\theta) = [Q_{A3}]_{y_r^-}^{y_r^+} &= 0 && \text{(S.109)}\\
Q_{A3}^{'}(y_\theta) = [Q_{A3}^{'}]_{y_r^-}^{y_r^+} &= (H_{ee}-1)n_{A3} + H_{ei}n_{I3} - \delta_e H_{ee}\partial_T X + D_e\Delta\nu_A \\
&\quad -2H_{ee}X\Big(H_{ee}\Pi_1 + H_{ei}\Pi_2 + D_e\Delta\nu_E\Big) + (H_{ee}^3 - H_{ee}^2)X^3 \\
&\quad +X\Big(H_{ee}\Pi_1 + H_{ei}\Pi_2 + D_e\Delta\nu_E\Big) + H_{ee}\Pi_1 X && \text{(S.110)}\\
\int_{-\infty}^{y_\theta} du Q_{A3}(u) &= 0 && \text{(S.111)}\\
Q_{B3}(y_\theta) = [Q_{B3}]_{y_r^-}^{y_r^+} &= 0 && \text{(S.112)}\\
Q_{B3}^{'}(y_\theta) = [Q_{B3}^{'}]_{y_r^-}^{y_r^+} &= (H_{ee}-1)n_{B3} + H_{ei}n_{I3} + \delta_e H_{ee}\partial_T X + D_e\Delta\nu_B \\
&\quad +2H_{ee}X\Big(H_{ee}\Pi_1 + H_{ei}\Pi_2 + D_e\Delta\nu_E\Big) - (H_{ee}^3 - H_{ee}^2)X^3 \\
&\quad -X\Big(H_{ee}\Pi_1 + H_{ei}\Pi_2 + D_e\Delta\nu_E\Big) - H_{ee}\Pi_1 X && \text{(S.113)}\\
\int_{-\infty}^{y_\theta} du Q_{B3}(u) &= 0 && \text{(S.114)}\\
Q_{I3}(\hat{y}_\theta) = [Q_{I3}]_{\hat{y}_r^-}^{\hat{y}_r^+} &= 0 && \text{(S.115)}\\
Q_{I3}(\hat{y}_\theta) = [Q_{I3}]_{\hat{y}_r^-}^{\hat{y}_r^+} &= H_{ie}(n_{A3} + n_{B3}) - n_{I3} && \text{(S.116)}\\
\int_{-\infty}^{\hat{y}_\theta} du Q_{I3}(u) &= 0 && \text{(S.117)}
\end{aligned}$$

The voltage distributions for populations A and B at this order are given by

$$Q_{A3}(y) = \begin{cases} \alpha_{A3}Q_E(y) + \beta_{A3}^{+}e^{-y^2} + Q_{A3}^{p0}(y) + Q_{A3}^{p1}(y) & y > y_r \\ \alpha_{A3}Q_E(y) + \beta_{A3}^{-}e^{-y^2} + Q_{A3}^{p0}(y) + Q_{A3}^{p1}(y) & y < y_r, \end{cases} \quad \text{(S.118)}$$

$$Q_{B3}(y) = \begin{cases} \alpha_{B3}Q_E(y) + \beta_{B3}^{+}e^{-y^2} + Q_{B3}^{p0}(y) + Q_{B3}^{p1}(y) & y > y_r \\ \alpha_{B3}Q_E(y) + \beta_{B3}^{-}e^{-y^2} + Q_{B3}^{p0}(y) + Q_{B3}^{p1}(y) & y < y_r, \end{cases} \quad \text{(S.119)}$$

It is not necessary to solve for $Q_{I3}(y)$.

One finds that

$$
\begin{aligned}
\alpha_{A3} &= n_{A3} + 2y_\theta Q^{p1}_{A3}(y_\theta) + \partial_y Q^{p1}_{A3}(y_\theta) + \delta_e H_{ee}\partial_T X - D_e\Delta\nu_A + X^3(H^2_{ee} - H^3_{ee}) \\
&\quad +2\Pi_1 X(H^2_{ee} - H_{ee}) + \Pi_2 X(2H_{ee}H_{ei} - H_{ei}) + (2H_{ee} - 1)D_e\Delta\nu_E X, \qquad \text{(S.120)} \\
\beta^+_{A3} &= -e^{y^2_\theta}Q^{p0}_{A3}(y_\theta) - e^{y^2_\theta}Q^{p1}_{A3}(y_\theta), \qquad \text{(S.121)} \\
\beta^-_{A3} &= \beta^+_{A3} + e^{y^2_r}[Q^{p0}_{A3}]^{y^+_r}_{y^-_r} + e^{y^2_r}[Q^{p1}_{A3}]^{y^+_r}_{y^-_r}, \qquad \text{(S.122)} \\
Q^{p0}_{A3} &= n_{A3}\left(- G_{ee}Q^{'}_E + \frac{1}{4}H_{ee}Q^{''}_E\right) + n_{I3}\left(G_{ei}Q^{'}_E + \frac{1}{4}H_{ei}Q^{''}_E\right), \qquad \text{(S.123)} \\
Q^{p1}_{A3} &= -XG_{ee}Q^{'}_{A2} + \frac{1}{4}XH_{ee}Q^{''}_{A2} + \Big(G_{ei}\Pi_2 - G_{ee}\Pi_1 - B_e\Delta\nu_E\Big)Q^{'}_{A1} \\
&\quad +\frac{1}{4}\Big(- 2X^2G_{ee} + H_{ee}\Pi_1 + H_{ei}\Pi_2 + D_e\Delta\nu_E\Big)Q^{''}_{A1} + \frac{1}{4}X^2G_{ee}H_{ee}Q^{'''}_{A1} - \frac{1}{32}X^2H^2_{ee}Q^{(iv)}_{A1} \\
&\quad +\Big(G_{ee}(\tau + \delta_e)\partial_T X - B_e\Delta\nu_A - \frac{S_A\xi_A}{\bar\sigma_E}\Big)Q^{'}_E \\
&\quad +\Big(- XG^2_{ee}\Pi_1 + XG_{ee}G_{ei}\Pi_2 - XG_{ee}B_e\Delta\nu_E + \frac{1}{4}D_e\Delta\nu_A - \frac{1}{4}(\frac{1}{2}\tau + \delta_e)H_{ee}\partial_T X\Big)Q^{''}_e \\
&\quad +\frac{1}{4}\Big(- \frac{2}{3}X^3G^3_{ee} + 2XG_{ee}H_{ee}\Pi_1 + X(G_{ee}H_{ei} - G_{ei}H_{ee})\Pi_2 + (H_{ee}B_e + G_{ee}D_e)\Delta\nu_E\Big)Q^{'''}_E \\
&\quad \frac{1}{16}\Big(2X^3G^2_{ee}H_{ee} - XH^2_{ee}\Pi_1 - XH_{ee}H_{ei}\Pi_2 - H_{ee}D_e\Delta\nu_E\Big)Q^{(iv)}_E - \frac{1}{32}X^3G_{ee}H^2_{ee}Q^{(v)}_E \\
&\quad +\frac{1}{384}X^3H^3_{ee}Q^{(vi)}_E + Q^T_{A3}(y)\tau\partial_T X, \qquad \text{(S.124)}
\end{aligned}
$$

where $\mathcal{L}Q^T_{A3} = Q_{A1} - Q^p_{A1}$, which can be integrated straightforwardly.

It is easily shown that $Q_{A3} = -Q_{B3}$ once the transformation $\Delta\nu_A \to -\Delta\nu_B$, $S_A\xi_A \to -S_B\xi_B$ has been made.

Once again the normalization conditions, Eqs.(S.111), (S.114) and (S.117), lead to a matrix equation, given by

$$\mathbb{L}\mathbf{n}_3 = \mathbf{N}_3, \qquad \text{(S.125)}$$

and once again we must have

$$\mathbf{n}^\dagger \cdot \mathbf{N}_3 = 0. \qquad \text{(S.126)}$$

Eq.(S.126) is the solvability condition which yields an equation for X given by

$$\tilde\tau\partial_T X = \hat\eta(\nu_{A,ext} - \nu_{B,ext}) + (\mu_E\Delta\nu_E + \mu_I\Delta\nu_I)X + \gamma X^3 + \bar\sigma\xi, \qquad \text{(S.127)}$$

where

$$\tilde{\tau} = 2\tau\Bigg\{\left[\frac{\sqrt{\pi}}{2}\left(G_{ee}(1+\frac{\delta_e}{\tau})+\frac{y}{4}H_{ee}(1+\frac{2\delta_e}{\tau})\right)e^{y^2}\mathrm{erfc}(-y)+Q_{A3}^T\right]_{y_r}^{y_\theta} - \frac{H_{ee}}{2\sqrt{\pi}\nu_E\tau}\Bigg\}, \quad \text{(S.128)}$$

$$\hat{\eta} = \Gamma(B_e, D_e), \quad \text{(S.129)}$$

$$\mu_E = \sqrt{\pi}\left[K_E(y)e^{y^2}\mathrm{erfc}(-y)\right]_{y_r}^{y_\theta} - 2\bar{K}_E, \quad \text{(S.130)}$$

$$\mu_I = \sqrt{\pi}\left[K_I(y)e^{y^2}\mathrm{erfc}(-y)\right]_{y_r}^{y_\theta} - 2\bar{K}_I, \quad \text{(S.131)}$$

$$\gamma = \sqrt{\pi}\left[K_C(y)e^{y^2}\mathrm{erfc}(-y)\right]_{y_r}^{y_\theta} - 2\bar{K}_C, \quad \text{(S.132)}$$

$$\bar{\sigma} = \sqrt{\frac{\pi}{2}}\frac{S_E}{\bar{\sigma}_E}\left[e^{y^2}\mathrm{erfc}(-y)\right]_{y_r}^{y_\theta}, \quad \text{(S.133)}$$

and

$$
\begin{aligned}
K_E(y) &= \Pi_{1E}\Bigg(2G_{ee} + yH_{ee} - 2yG_{ee}^2 - (1+2y^2)G_{ee}H_{ee} - (\frac{3y}{4} + \frac{y^3}{2})H_{ee}^2\Bigg) \\
&\quad \Pi_{2E}\Bigg(- G_{ei} + \frac{y}{2}H_{ei} + 2yG_{ee}G_{ei} + (\frac{1}{2} + y^2)(G_{ei}H_{ee} - G_{ee}H_{ei}) \\
&\quad -(\frac{3y}{4} + \frac{y^3}{2})H_{ee}H_{ei}\Bigg) + \Bigg(1 - 2yG_{ee} - (\frac{1}{2} + y^2)H_{ee}\Bigg)B_e \\
&\quad \Bigg(\frac{y}{2} - (\frac{1}{2} + y^2)G_{ee} - (\frac{3y}{4} + \frac{y}{2})H_{ee}\Bigg)D_e, \qquad \text{(S.134)}
\end{aligned}
$$

$$
\begin{aligned}
\bar{K}_E(y) &= \Pi_{1E}\Bigg((y_\theta - y_r)G_{ee}H_{ee} + \frac{(y_\theta^2 - y_r^2)}{4}H_{ee}^2\Bigg) \\
&\quad +\Pi_{2E}\Bigg(\frac{(y_\theta - y_r)}{2}(G_{ee}H_{ei} - G_{ei}H_{ee}) + \frac{(y_\theta^2 - y_r^2)}{4}H_{ee}H_{ei}\Bigg) \\
&\quad +\frac{(y_\theta - y_r)}{2}H_{ee}B_e + \Bigg(\frac{(y_\theta - y_r)}{2}G_{ee} + \frac{(y_\theta^2 - y_r^2)}{4}H_{ee}\Bigg)D_e \qquad \text{(S.135)}
\end{aligned}
$$

$$
K_I(y) = \Pi_{1I}\Bigg(2G_{ee} + yH_{ee} - 2yG_{ee}^2 - (1+2y^2)G_{ee}H_{ee} - (\frac{3y}{4} + \frac{y^3}{2})H_{ee}^2\Bigg) \qquad \text{(S.136)}
$$

$$
\bar{K}_{LI}(y) = \Pi_{1I}\Bigg((y_\theta - y_r)G_{ee}H_{ee} + \frac{(y_\theta^2 - y_r^2)}{4}H_{ee}^2\Bigg), \qquad \text{(S.137)}
$$

$$
\begin{aligned}
K_C(y) &= \Pi_{1C}\Bigg(2G_{ee} + yH_{ee} - 2yG_{ee}^2 - (1+2y^2)G_{ee}H_{ee} - (\frac{3y}{4} + \frac{y^3}{2})H_{ee}^2\Bigg) \\
&\quad +\Pi_{2C}\Bigg(- G_{ei} + \frac{y}{2}H_{ei} + 2yG_{ee}G_{ei} + (\frac{1}{2} + y^2)(G_{ei}H_{ee} - G_{ee}H_{ei}) \\
&\quad -(\frac{3y}{4} + \frac{y^3}{2})H_{ee}H_{ei}\Bigg) - yG_{ee}^2 - (\frac{3y}{8} + \frac{y^3}{4})H_{ee}^2 - (\frac{1}{2} + y^2)G_{ee}H_{ee} \\
&\quad +(\frac{3}{8} + \frac{3y^2}{2} + \frac{y^4}{2})G_{ee}H_{ee}^2 + (\frac{3y}{2} + y^3)G_{ee}^2H_{ee} + (\frac{1}{3} + \frac{2y^2}{3})G_{ee}^3 \\
&\quad +(\frac{5y}{16} + \frac{5y^3}{12} + \frac{y^5}{12})H_{ee}^3, \qquad \text{(S.138)}
\end{aligned}
$$

$$
\begin{aligned}
\bar{K}_C(y) &= \Pi_{1C}\Bigg((y_\theta - y_r)G_{ee}H_{ee} + \frac{(y_\theta^2 - y_r^2)}{4}H_{ee}^2\Bigg) \\
&\quad +\Pi_{2C}\Bigg(\frac{(y_\theta - y_r)}{2}(G_{ee}H_{ei} - G_{ei}H_{ee}) + \frac{(y_\theta^2 - y_r^2)}{4}H_{ee}H_{ei}\Bigg) \\
&\quad +\frac{(y_\theta - y_r)}{2}G_{ee}H_{ee} + \frac{(y_\theta^2 - y_r^2)}{8}H_{ee}^2 - \frac{(y_\theta - y_r)}{3}G_{ee}^3 \\
&\quad -\frac{(y_\theta^2 - y_r^2)}{2}G_{ee}^2H_{ee} - \frac{1}{4}\Big(\frac{5(y_\theta - y_r)}{2} + y_\theta^3 - y_r^3\Big)G_{ee}H_{ee}^2 \\
&\quad +\frac{1}{8}\Big(\frac{3(y_\theta^2 - y_r^2)}{2} + \frac{(y_\theta^4 - y_r^4)}{3}\Big)H_{ee}^3. \qquad \text{(S.139)}
\end{aligned}
$$

Finally, if we assume that $\Delta\nu_I = 0$, i.e. the input to the inhibitory neurons is fixed, we can rewrite Eq.S.127 in the form of Eq.S.1 where $\eta = \hat{\eta}\frac{\gamma^{1/2}}{\tau^{3/2}}$, $\mu = \frac{\mu_E}{\tilde{\tau}}$ and $\sigma = \bar{\sigma}\frac{\gamma^{1/2}}{\tau^{3/2}}$.

2.1.7 Comparison with numerical simulations of full system

We now, as with the rate equations, compare the quantitative match between the network model and Eq.S.127. Fig.S.3 shows bifurcation diagrams for the case of a supercritical bifurcation (top left), a subcritical bifurcation (top right), and an imperfect supercritical bifurcation (bottom left). Note that these three bifurcation diagrams provide confirmation of the quantitative accuracy of the steady-state prediction from Eq.S.127. The transient dynamics for the imperfect, subcritical case are compared on the bottom right. This is the case of interest for us. As can be seen, the agreement between simulations of the full network and Eq.S.127 is not as good as for the rate equations. As mentioned, the region of quantitative agreement will depend on the system of departure.

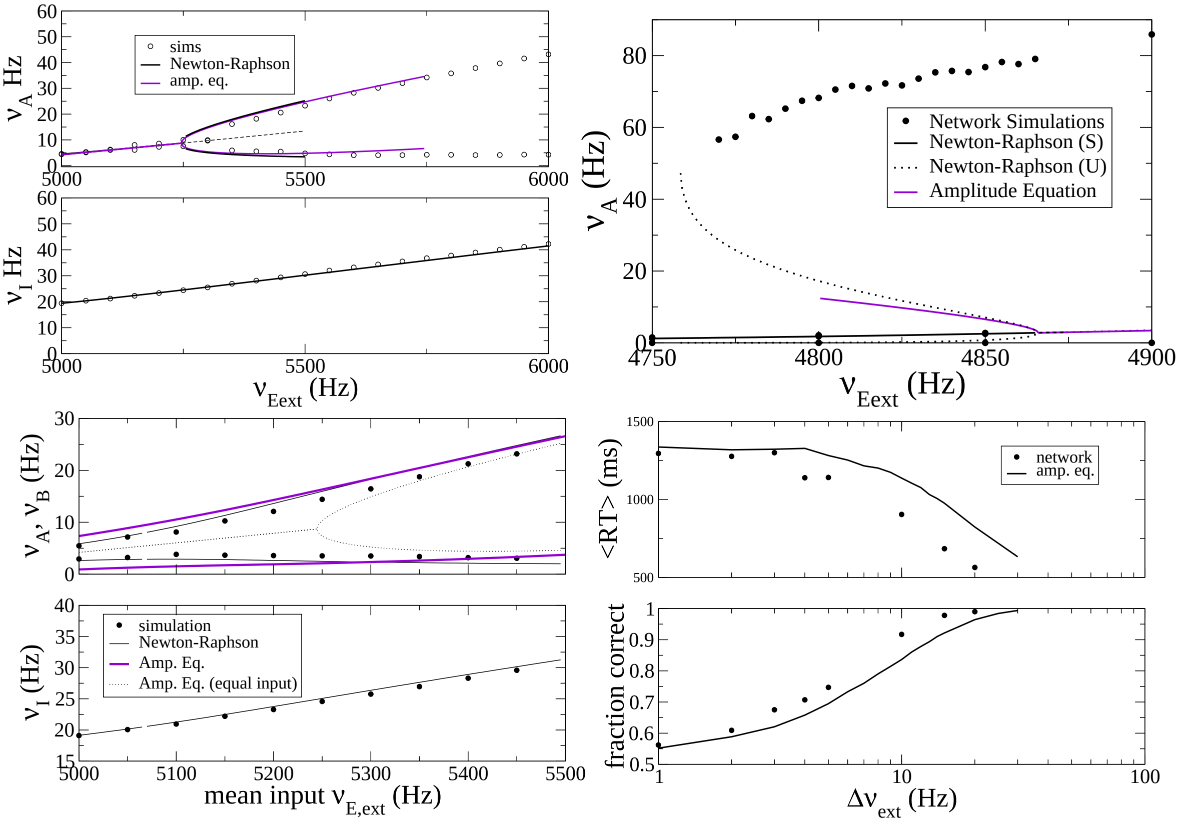

Fig. S.3: Comparison of spiking network and amplitude equation. Upper left: Supercritical bifurcation. $J_{ee} = 0.1$mV. Upper right: Subcritical bifurcation. $J_{ee} = 0.16$mV, $J_{ei} = 0.22$mV. Lower left: Imperfect supercritical bifurcation. Difference in inputs between the two populations was 50Hz. Lower Right: Mean reaction times and performance as a function of the difference in presynaptic firing rates. $J_{ee} = 0.16$ and $J_{ei} = 0.20$. The mean input to both populations was 4790Hz where the bifurcation occurs at 4792.5Hz. Unless otherwise noted parameter values were: $N_e = 1000$, $N_i = 500$, $C_{ee} = 100$, $C_{ei} = C_{ie} = C_{ext} = 50$, $J_{ext} = 0.2$mV, $J_{ie} = 0.1$mV, $J_{ei} = 0.2$mV, $\tau = 20$ms, $\hat{\tau} = 10$ms, $E_e = E_i = -70$mV, $V_r = \hat{V}_r = -60$mV, $V_\theta = \bar{V}_\theta = -50$mV. The excitatory (inhibitory) synaptic delays were uniformly distributed with a mean of 5ms (1ms).

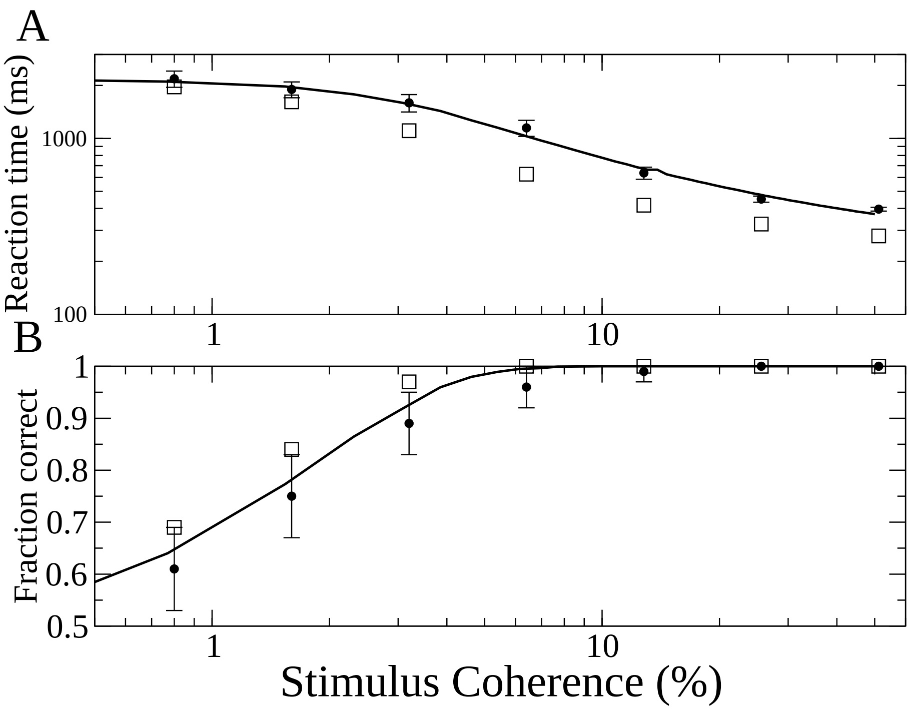

Fig. S.4: Comparison of network of integrate-and-fire network (open squares) with Eq.S.127 (solid line). We have used the fit of Eq.S.127 to behavioral data (filled circles with error bars) from Fig.4c and 4d of the main text. See methods for parameter values. The parameter values used are : $E_e = E_i = -70$mV, $V_r = \hat{V}_r = -60$mV, $\theta = \hat{\theta} = -50$mV, $\tau = 20$ms, $\hat{\tau} = 10$mV, $J_{ee} = 0.16$mV, $J_{ie} = 0.1$mV, $J_{ei} = 0.2$mV, $J_{ext} = 0.20$ $N_E = 2000$, $N_I = 1000$, $C_{ee} = 100$, $C_{ie} = 50$, $C_{ei} = 50$. Excitatory (inhibitory) delays were taken from a uniform distributions with a mean of 5ms (0.5ms). The form of this distribution (as long as delays are not too long) does not affect the reaction-time dynamics qualitatively. The external Poisson rate for the inhibitory population was held fixed at 9000 Hz, e.g. 90 external synapses firing at 10 Hz each. Those for the excitatory populations where held at 4750 Hz for 200 ms and then increased to $4790 + 1.425 * \text{coherence}$ Hz.

2.2 Example 2: Reduced equations from Wong and Wang 2006

The equations are

$$\dot{s}_1 = -\frac{s_1}{\tau_s} + (1-s_1)H\Big(J_e s_1 - J_i s_2 + I_0 + I_1 + I_{\text{noise},1}(t)\Big), \quad \text{(S.140)}$$

$$\dot{s}_s = -\frac{s_s}{\tau_s} + (1-s_s)H\Big(J_e s_s - J_i s_1 + I_0 + I_2 + I_{\text{noise},2}(t)\Big), \quad \text{(S.141)}$$

where the noisy inputs obey

$$\tau_{\text{ampa}}\dot{I}_{\text{noise},i} = -I_{\text{noise},i} + \sigma\sqrt{\tau_{\text{ampa}}}\eta_i(t), \quad \text{(S.142)}$$

and $H(I) = \gamma\frac{(aI-b)}{1-e^{-d(aI-b)}}$. The derivation of these equations from the full spiking network can be found in [11].

2.2.1 Linear Stability

We assume that $I_1 \sim I_2$ and consider an ansatz of the form $(s_A, s_B) = (\bar{s}, \bar{s}) + (\delta s_1, \delta s_2)e^{\lambda t}$, where $\bar{s} = (1-\bar{s})H(\bar{s}(J_e - J_i) + I_0)$. This leads to an eigenvalue problem of the form

$$\begin{pmatrix} \lambda + \frac{1}{\tau_s} + H - J_e(1-\bar{s})H^{'} & J_i(1-\bar{s})H^{'} \\ J_i(1-\bar{s})H^{'} & \lambda + \frac{1}{\tau_s} + H - J_e(1-\bar{s})H^{'} \end{pmatrix} \begin{pmatrix} \delta r_A \\ \delta r_B \\ \delta r_I \end{pmatrix} = 0, \quad \text{(S.143)}$$

where H and its derivatives are evaluated at the fixed point.

The eigenvalue corresponding to the eigenvector $(1,-1)$ is equal to zero for $\frac{1}{\tau_s} + H - J_e(1-\bar{s})H^{'} = J_i(1-\bar{s})H^{'}$, which occurs for $I_0 = I_{cr}$.

2.2.2 Weakly Nonlinear Dynamics

We expand around the steady instability found above. We take

$$I_1 = \epsilon^2\Delta I_0 + \hat{\epsilon}\Delta I_1 \quad \text{(S.144)}$$

$$I_2 = \epsilon^2\Delta I_0 + \hat{\epsilon}\Delta I_2 \quad \text{(S.145)}$$

$$(s_1, s_2) = (\bar{s}, \bar{s}) + \epsilon(1,-1)X(T) + \epsilon^2(s_{12}, s_{22}) + \ldots, \quad \text{(S.146)}$$

where ϵ and $\hat{\epsilon}$ are small parameters which measure the distance from the bifurcation and the difference in inputs to the two excitatory populations respectively. Near the bifurcation, the mode corresponding to the critical eigenvector $X(T)$ evolves on the slow time scale $T = \epsilon^2 t$. The expansions given above are plugged into

Eqs(S.140-S.141) and terms are collected order by order. We assume that $\hat{\epsilon} = \vartheta(\epsilon^3)$.

$\vartheta(\epsilon)$:

We recover the linear stability problem

$$\mathbf{L s_1} = 0, \tag{S.147}$$

where

$$\mathbf{L} = J_i(1-\bar{s})H^{'} \begin{pmatrix} 1 & 1 \\ 1 & 1 \end{pmatrix} \tag{S.148}$$

and $\mathbf{s_1} = (1,-1)X(T)$.

$\vartheta(\epsilon^{\mathbf{2}})$:

$$\begin{aligned} \mathbf{L r_2} &= \mathbf{N_2}, && \text{(S.149)} \\ \mathbf{N_2} &= \Big((1-\bar{s})H^{'}\Delta I_0 + (1-\bar{s})\frac{H^{''}}{2}(J_e+J_i)^2X^2 \\ &\quad -H^{'}(J_e+J_i)X^2\Big)\begin{pmatrix} 1 \\ 1 \end{pmatrix}. && \text{(S.150)} \end{aligned}$$

We solve for s_2 as in section 2.1,

$$\mathbf{s_2} = \mathcal{S}\begin{pmatrix} 1 \\ 1 \end{pmatrix}, \tag{S.151}$$

where

$$\mathcal{S} = \frac{1}{2J_i}\Delta I_0 + \left[\frac{H^{''}(J_e+J_i)^2}{4J_iH^{'}} - \frac{(J_e+J_i)}{2(1-\bar{s})J_i}\right]X^2 \tag{S.152}$$

$\vartheta(\epsilon^{\mathbf{3}})$:

We have

$$\mathbf{L r_3} + \mathbf{L_2 r_1} = \mathbf{N_3}, \tag{S.153}$$

where

$$\begin{aligned} \mathbf{L_2} &= \begin{pmatrix} \partial_T + (H^{'} - (1-\bar{s})H^{''}J_e)\Delta I_0 & (1-\bar{s})H^{''}J_i\Delta I_0 \\ (1-\bar{s})H^{''}J_i\Delta I_0 & \partial_T + (H^{'} - (1-\bar{s})H^{''}J_e)\Delta I_0 \end{pmatrix}, && \text{(S.154)} \\ \mathbf{N_3} &= \mathcal{N}_3\begin{pmatrix} 1 \\ -1 \end{pmatrix} + \begin{pmatrix} (1-\bar{s})H^{'}\Delta I_1 \\ (1-\bar{s})H^{'}\Delta I_2 \end{pmatrix} && \text{(S.155)} \end{aligned}$$

where

$$\begin{aligned}
\mathcal{N}_3 &= \frac{1}{2J_i}\left((1-\bar{s})H^{''}(J_e^2-J_i^2)-2J_eH^{'}\right)\Delta I_0 X \\
&+\left[\left((1-\bar{s})H^{''}(J_e^2-J_i^2)-2H^{'}J_e\right)\left(\frac{H^{''}(J_e+J_i)^2}{4J_iH^{'}}-\frac{(J_e+J_i)}{2(1-\bar{s})J_i}\right)+\frac{(1-\bar{s})}{6}H^{''}(J_e+J_i)^3\right. \\
&\left.-\frac{H^{''}}{2}(J_e+J_i)^2\right]X^3 \qquad \text{(S.156)}
\end{aligned}$$

Again, Eq.(S.153) only has a solution if $\langle \mathbf{s}^{\dagger}, \mathbf{L_2 s_1} - \mathbf{N_3}\rangle = 0$, where $\mathbf{s}^{\dagger} = (1, -1)$. This leads to the equation

$$\partial_T X = \eta(I_1 - I_2) + \mu\Delta I_0 X + \gamma X^3 + \hat{\sigma} I_{\text{noise}}, \qquad \text{(S.157)}$$

where

$$\eta = \frac{(1-\bar{s})H^{'}}{2} \qquad \text{(S.158)}$$

$$\mu = (J_e+J_i)(1-\bar{s})H^{''} - H^{'} + \frac{(J_e^2-J_i^2)(1-\bar{s})H^{''}}{2J_i} - \frac{J_eH^{''}}{J_i} \qquad \text{(S.159)}$$

$$\begin{aligned}
\gamma &= \left((1-\bar{s})H^{''}(J_e^2-J_i^2)-2J_eH^{'}\right)\left(\frac{H^{''}(J_e+J_i)^2}{4J_iH^{'}}-\frac{(J_e+J_i)}{2(1-\bar{s})J_i}\right) \\
&+\frac{(1-\bar{s})H^{'''}(J_e+J_i)^3}{6}-\frac{H^{''}(J_e+J_i)^2}{2} \qquad \text{(S.160)}
\end{aligned}$$

$$\hat{\sigma} = \frac{(1-\bar{s})H^{'}}{\sqrt{2\tau_s}} \qquad \text{(S.161)}$$

$$\tau_{\text{ampa}}\dot{I}_{\text{noise}} = -I_{\text{noise}} + \sigma\sqrt{\tau_{\text{ampa}}}\eta(t) \qquad \text{(S.162)}$$

Fig.S.5 shows the bifurcation diagram of the original system (black lines) and the estimation of the fixed points near the bifurcation given by Eq.S.157 (red lines). Fig.S.6 shows a sample simulation of the full system (black) and the nonlinear diffusion equation (red) using the same seed to generate the random fluctuations. The simultation was done at the bifurcation point for symmetric inputs.

Note that in this example as in the previous one (network of spiking neurons) the noise is both additive and multiplicative in the original equations. Assuming small-amplitude noise results in the approximation of purely additive noise. As the noise strength is increased this approximation breaks down. The multiplicative noise can be takin into account in the amplitude equation by retaining higher order terms: the $\vartheta(\epsilon^4)$ term $\hat{\mu}(t)X$, where $\hat{\mu}(t)$ is proportional to the noise. For consistency one must then include the $\vartheta(\epsilon^4)$ term bX^2 as well.

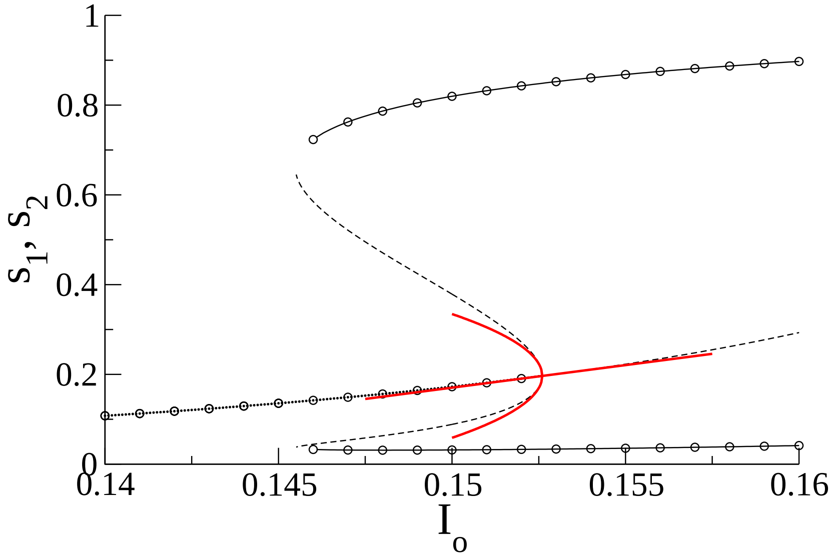

Fig. S.5: Bifurcation diagram for the reduced model, Eqs.S.140-S.141. Parameter values are $a = 173.07$, $b = 108$, $d = 0.154$, $\tau_s = 100$, $J_e = 0.2609$, $J_i = 0.0497$ $I_1 = I_2 = 0$. Black lines: stable (solid) and unstable (dashed) branches calculated with a Newton-Raphson scheme. Circles: Numerical simulation of Eqs.S.140-S.141. Red lines: amplitude equation solution Eq.S.157.

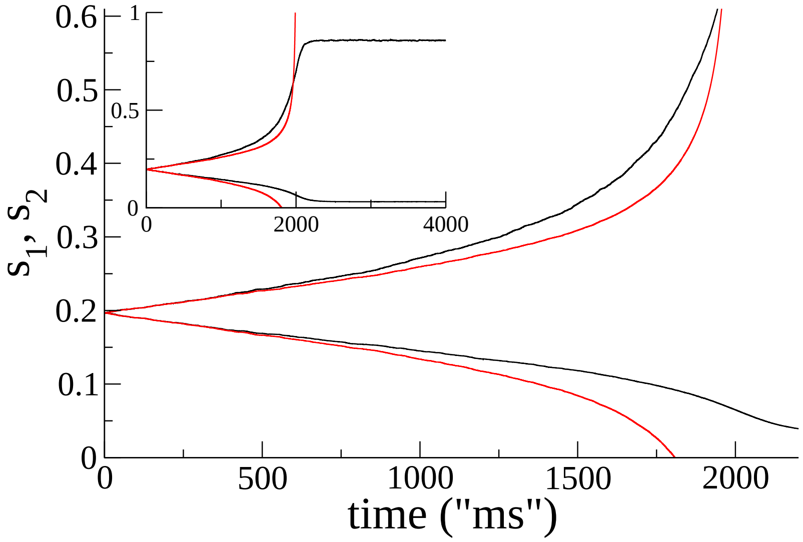

Fig. S.6: Sample traces of $s_1(t)$ and $s_2(t)$ (black lines) and $X(T)$ (red lines) from a single numerical realization. The same seed is used for generating the noise in both cases in order to compare the actual temporal evolution. Parameter values are as in Fig.S.6 with $I_0 = 0.1526$ (at the bifurcation point), $I_1 = 0.00081$, $I_2 = -0.00081$, $\tau_{\rm ampa} = 2$, $\sigma = 0.001$.

References

[1] N. Brunel. Dynamics of sparsely connected networks of excitatory and inhibitory spiking neurons. *J. Comput. Neurosci.*, 8:183–208, 2000.

[2] N. Brunel and V. Hakim. Fast global oscillations in networks of integrate-and-fire neurons with low firing rates. *Neural Comp.*, 11:1621–1671, 1999.

[3] P. Glendinning. *Stability, instability and chaos.* Cambridge: Cambridge University Press, 1994.

[4] J. Guckenheimer and P. Holmes. *Nonlinear oscillations, dynamical systems, and bifurcations of vector fields.* Springer Verlag, 1983.

[5] H. Haken. *Synergetics.* Springer Verlag, 1983.

[6] Samuel Karlin and Howard M. Taylor. *A second course in stochastic processes.* Academic Press, New York, first edition, 1981.

[7] J. Palmer, A. C. Huk, and M. N. Shadlen. The effect of stimulus strength on the speed and accuracy of a perceptual decision. *J. of Vision*, 5:376–404, 2005.

[8] S. H. Strogatz. *Nonlinear Dynamics and Chaos.* Addison Wesley Publishing Company, 1994.

[9] X.-J. Wang. Probabilistic decision making by slow reverberation in cortical circuits. *Neuron*, 36:955–968, 2002.

[10] S. Wiggins. *Introduction to Applied Nonlinear Dynamical Systems and Chaos.* Springer, 2nd edition, 2003.

[11] K. F. Wong and X-J Wang. A recurrent network mechanism of time integration in perceptual decisions. *J. of Neurosci.*, 26(4):1314–1328, 2006.
